# Integrated analysis identifies microRNA-195 as a suppressor of Hippo-YAP pathway in colorectal cancer

**DOI:** 10.1186/s13045-017-0445-8

**Published:** 2017-03-29

**Authors:** Min Sun, Haibin Song, Shuyi Wang, Chunxiao Zhang, Liang Zheng, Fangfang Chen, Dongdong Shi, Yuanyuan Chen, Chaogang Yang, Zhenxian Xiang, Qing Liu, Chen Wei, Bin Xiong

**Affiliations:** Department of Oncology, Zhongnan Hospital of Wuhan University, Hubei Key Laboratory of Tumor Biological Behaviors & Hubei Cancer Clinical Study Center, 430071 Wuhan, People’s Republic of China

**Keywords:** Hsa-miRNA-195(miR-195), YAP1, Colorectal cancer (CRC), Epithelial mesenchymal transition (EMT), Prognosis, Biomarker

## Abstract

**Background:**

With persistent inconsistencies in colorectal cancer (CRC) miRNAs expression data, it is crucial to shift toward inclusion of a “pre-laboratory” integrated analysis to expedite effective precision medicine and translational research. Aberrant expression of hsa-miRNA-195 (miR-195) which is distinguished as a clinically noteworthy miRNA has previously been observed in multiple cancers, yet its role in CRC remains unclear.

**Methods:**

In this study, we performed an integrated analysis of seven CRC miRNAs expression datasets. The expression of miR-195 was validated in The Cancer Genome Atlas (TCGA) datasets, and an independent validation sample cohort. Colon cancer cells were transfected with miR-195 mimic and inhibitor, after which cell proliferation, colony formation, migration, invasion, and dual luciferase reporter were assayed. Xenograft mouse models were used to determine the role of miR-195 in CRC tumorigenicity in vivo.

**Results:**

Four downregulated miRNAs (hsa-let-7a, hsa-miR-125b, hsa-miR-145, and hsa-miR-195) were demonstrated to be potentially useful diagnostic markers in the clinical setting. CRC patients with a decreased level of miR-195-5p in tumor tissues had significantly shortened survival as revealed by the TCGA colon adenocarcinoma (COAD) dataset and our CRC cohort. Overexpression of miR-195-5p in DLD1 and HCT116 cells repressed cell growth, colony formation, invasion, and migration. Inhibition of miR-195-5p function contributed to aberrant cell proliferation, migration, invasion, and epithelial mesenchymal transition (EMT). We identified miR-195-5p binding sites within the 3’-untranslated region (3′-UTR) of the human yes-associated protein (YAP) mRNA. YAP1 expression was downregulated after miR-195-5p treatment by qRT-PCR analysis and western blot.

**Conclusions:**

Four downregulated miRNAs were shown to be prime candidates for a panel of biomarkers with sufficient diagnostic accuracy for CRC in a clinical setting. Our integrated microRNA profiling approach identified miR-195-5p independently associated with prognosis in CRC. Our results demonstrated that miR-195-5p was a potent suppressor of YAP1, and miR-195-5p-mediated downregulation of YAP1 significantly reduced tumor development in a mouse CRC xenograft model. In the clinic, miR-195-5p can serve as a prognostic marker to predict the outcome of the CRC patients.

**Electronic supplementary material:**

The online version of this article (doi:10.1186/s13045-017-0445-8) contains supplementary material, which is available to authorized users.

## Background

Colorectal cancer (CRC) has been the fourth leading cause of cancer death worldwide for several decades [1]. The 5-year survival rate for localized stage CRC is 90%, whereas as CRC spreads to the regional lymph nodes or distant parts of the body, the 5-year survival rate plunges from 71% to 13% in the United States [2]. These malignancies are ascribed to accumulation of genetic alterations, including dissemination of proto-oncogenes, losing or inactivating of tumor suppressor genes, and EMT, which ultimately lead to tumor evolution and progression [3–7].

Using the miRNA expression profile, dysregulated microRNAs (miRNA/miR) in CRC can be identified. Specific miRNAs have been suggested as biomarkers for CRC pathogenesis, metastasis, recurrence, and as potential therapeutic targets [8]. Previous research, by examining a series of CRC microRNAs expression profile data, recommended several deregulated miRNAs, but most failed to identify efficient miRNA biomarkers. Furthermore, few mechanistic studies address the question of how miRNA drives the alterations of the hallmark CRC signaling pathways [9]. For a collective set of deregulated miRNAs, experimental validation is still lacking, and the potential mRNA targets of these miRNAs and their regulatory mechanisms remain unknown. The multicenter sequencing of 1893 carcinoma/normal paired samples revealed that miR-195-5p was a tumor-suppressing miRNA and was downregulated in tumor tissues [10]. Since predicted targets of miR-195-5p were enriched for the Hippo/YAP signaling pathway, we hypothesize that miR-195-5p may function by targeting the *YAP1* mRNA. Recent studies showed that YAP and its transcriptional coactivator with PDZ-binding motif (TAZ) function as a nexus on the crosstalk of multiple signaling pathways, and therefore can be potential therapeutic targets as the chief downstream effectors of the Hippo pathway in a variety of cancers [11–15]. It was documented that YAP and TAZ overexpression resulted in a trend of shorter survival time by multivariate analysis in CRC [16]. However, there have been few studies on miRNAs that regulate the oncogenic alterations in the Hippo signaling pathway.

We performed an integrated analysis and identified significantly disturbed miRNAs by using microRNA sequencing data in primary tumors and paired adjacent normal tissue (PANT), both derived from the CRC patients. We further validated aberrant expression patterns of these miRNAs in the TCGA-COAD dataset [17]. We distinguished miR-195-5p as a clinically noteworthy miRNA in CRC as found in lung cancer [18]. We confirmed that the miR-195-5p level was high in patients with favorable survival in TCGA, and may play a critical role in CRC tumorigenesis. Ectopic expression of miR-195-5p could significantly reduce proliferation, migration, invasion, and EMT in two colon cancer cell lines, DLD1 and HCT116. We showed for the first time that the full-length 3′-UTR of the human *YAP1* mRNA was a direct target of miR-195-5p. These data offer a plausible mechanism accounting for the tumor-suppressing function of miR-195-5p, further supporting the notion that the miR-195-5p/YAP1/EMT axis can be the therapeutic focus for eliminating CRC tumorigenesis [19].

## Methods

### Search strategy and data collection, preprocessing, and normalization

Public microarray repositories were curated to search through the PubMed, GEO (accession numbers GSE33124, GSE35982, GSE33122, GSE35834, GSE33125, GSE33123, GSE68306, GSE35834, GSE41655, GSE41012, GSE73178, GSE54632, GSE68377, GSE48267, GSE54088, GSE45349, GSE38389, GSE28364, GSE7828, GSE35602, GSE10259 and GSE49246) and Array Express (accession numbers E-MTAB-96, E-GEOD-35834, E-GEOD-28364 and E-MTAB-2479) through September 2015 (the full-detailed search strategy and search terms are shown in Additional file 1: Table S1). The reference sections of the regained studies were analyzed for additional pertinent papers. The checklist and pipeline for properly organizing the integrated analysis were decided as recommended by Ramasamy, via the reporting guidelines of microarray meta-analysis [20]. Only original experimental studies that screened for different miRNAs between CRC and PANT in humans were the first choice for inclusion. Additional criteria for selection dictated that the included datasets should contain at least 30 samples of CRC and PANT correspondingly. Exclusion criteria included: (1) repeated reports by the same institute, hospital; (2) a non-expression gene chip; and (3) a non-whole-genome chip. All datasets were normalized individually on the base-2 logarithm by Robust Multi-Array Average (RMA) and Linear Models for Microarray (LIMMA) package and annotated by converting different probe IDs to gene IDs. All miRNA names were standardized according to miRBase version 17 via miRBase Tracker and consisted with TCGA for subsequent validation [21]. Any probe that did not map to a gene ID was removed as viral miRNAs or non-miRNA probes.

### Integrated analysis of miRNAs expression datasets

To identify differentially expressed (DE) miRNAs between CRC and PANT, we used MetaOmics software (http://www.pitt.edu/~tsengweb/MetaOmicsHome.htm) executed as a MetaDE package [22]. For integrated analysis, the mean and standard deviation (SD) filter thresholds were specified as 10%. Considering the different stringencies of the methods, Fisher’s method was performed for statistical analysis of significance: a modified *t* test and permutation method were used to extrapolate the *P* values [23]. One-sided tests were applied to classify the upregulated or downregulated DE miRNAs. A *P* value less than 0.05 was considered statistically significant for the DE miRNAs.

### Integrated-signature miRNA analysis of TCGA

The results of integrated analysis of miRNAs expression datasets were validated in the TCGA datasets. TCGA-COAD miRNA data and clinical data (level 3) of the corresponding patients (tumor and/or adjacent normal tissue) were downloaded from the TCGA Data portal (up to May 20, 2016). The expression analyses were carried out using BRB-ArrayTools (version 4.5, National Cancer Institute, Bethesda, MD, USA) [24].

### Target prediction and functional analysis of miRNA

The presumed targets of integrated-signature miRNAs, especially the most significant has-miR-195, were identified by four different target prediction algorithms: TargetScan v7.1 (http://www.targetscan.org/vert_71/), miRDB (http://www.mirdb.org/miRDB/), DIANA-TarBase v7.0 (http://diana.imis.athena-innovation.gr/DianaTools/index.php?r=tarbase/index/), and PicTar (http://pictar.mdc-berlin.de/cgi-bin/PicTar_vertebrate.cgi). Unique genes with target sites in 3′UTR were incorporated. To assess the prospective functions of the most significant has-miR-195, we discharged the Kyoto Encyclopedia of Genes and Genomes (KEGG) using the Database for Annotation, Visualization and Integrated Discovery (DAVID) [25]. The *P* value that narrated KEGG pathway enrich the target gene less than 0.05 was defined as the cutoff criterion.

### Tissue collection

Sixty human CRC tissues and PANT (distance to cancer > 5 cm) were acquired from patients who had been diagnosed with primary CRC by pathological assessment of tissues and undergone surgeries with complete prognostic information at the Zhongnan Hospital of Wuhan University between January 2011 and December 2015. No local or systemic neoadjuvant radiotherapy, or/and chemotherapy and targeted therapy were managed. The study was approved by the Research Ethics Committee of Wuhan University (Wuhan, Hubei, PR China). Informed consents were obtained from all participating patients.

### Cell culture and transfection of miR-195 mimic, inhibitor, and siRNA of target gene

Human colon cancer cell lines DLD1 and HCT116, and normal intestinal epithelium cell line NCM460 were purchased from the Cell Bank of Wuhan University. Cells were cultured in DMEM medium (Gibco, USA) containing 2 mmol/L glutamine, 10% heat-inactivated (56 °C, 30 min) fetal calf serum, streptomycin (100 U/mL) and penicillin (100 U/mL), and maintained in a humidified atmosphere of 5% CO_2_ at 37 °C. Hsa-miR-195 mimic and mimic negative control (NC), hsa-miR-195 inhibitor, and inhibitor negative control (NC) were purchased from RiboBio Co., Ltd. (Guangzhou, China). Cells were cultured in complete medium at least 24 h before transfection. Cells were washed by phosphate-buffered saline (PBS, pH 7.4) before transient transfection with 50 nM miR-195-5p mimic or miR mimic NC, 100 nM miR-195-5p inhibitor or miR inhibitor NC. Three siRNA duplexes targeting human YAP1 (GenBank accession no. NM_001130145.2) were synthesized by RiboBio Company (Guangzhou, China). Transfections were performed by Lipofectamine 2000 (Invitrogen, USA) according to the manufacturer’s protocol with RNA oligonucleotides at a final concentration of 50 nM.

### Total RNA extraction and quantitative reverse transcription polymerase chain reaction (qRT-PCR)

Total RNA was extracted using a Trizol reagent (Invitrogen). For the qRT-PCR detection of mature miR-195-5p expression, we purchased the Bulge-Loop™ miRNA qRT-PCR Primer Set and Control Primer Set (RiboBio, Guangzhou, China). RNA (2 μg) was converted into cDNA using the RevertAid First Strand cDNA Synthesis Kit (Thermo). QRT-PCR was accomplished using the FastStart Universal SYBR Green Master (Rox) (Roche) in the ABI PRISM^®^ 7300 real-time PCR system (Applied Biosystems, Foster City, CA, USA). GADPH and U6 were used as endogenous controls. We used melting curves to monitor non-specific amplifications. Relative expression level was computed using 2^-ΔΔCt^ method. The primer sequences used are listed in Supplemental Experimental Procedures.

### Tumor formation in BALB/c nude mice and immunohistochemistry

BALB/c athymic nude mice (female, 4 – 6 weeks old and 16 – 20 g) were purchased from Hubei Research Center of Laboratory Animals (Wuhan, China). All animal experiments were carried out in accordance with the Guide for the Care and Use of Laboratory Animals of Wuhan University. To establish the CRC cancer xenograft model, 5 × 10^6^ DLD1 cells were suspended in 150 μL PBS and inoculated subcutaneously into the flanks of the nude mice. After 8 days, the transplanted nude mice were randomly divided into two groups (*n* = 6 each). MiR-195-5p agomir or miR agomir NC (RiboBio Co., Ltd, Guangzhou, China) was directly injected into the implanted tumor at the dose of 2 nmol/30μL PBS per mouse every 4 days for seven times. Tumor dimension was measured by length (L) and width (W) with a caliper every 4 days, and the volumes were calculated using the formula: (L × W^2^)/2. Mice were sacrificed by cervical dislocation after being anaesthetized with 10% chloral hydrate at day 36, and the tumors were excised and snap-frozen for protein and RNA extraction. Immunohistochemistry of the tumor tissues was performed as described previously [26].

### Statistical analysis

The significance of the differences between the groups was determined with an ANOVA test or Student’s *t* test, and *P* < 0.05 was considered statistically significant. The results were expressed as the mean ± SD from at least three independent experiments. Kaplan-Meier method and Cox's proportional hazards regression model were used to calculate overall survivals, and the differences were analyzed by a log-rank test. Receiver operating characteristic (ROC) curve analysis was performed using the code of Mihaly (Additional file 2: Supplemental R script) by R Software (version 3.2.3; http://www.r-project.org) [27].

Supplemental experimental procedures include the following information: dual luciferase reporter assays, BrdU immunofluorescence assay, colony formation assay, CCK8 assay, wound healing assay in vitro, transwell migration/invasion assay, transfection reagents, primers, western blot analysis, and antibody (Additional file 3: Supplemental Experimental Procedures).

## Results

### Integrated analysis of seven CRC miRNAs expression datasets identified 21 significantly deregulated miRNAs in CRC

**Fig. 1:**
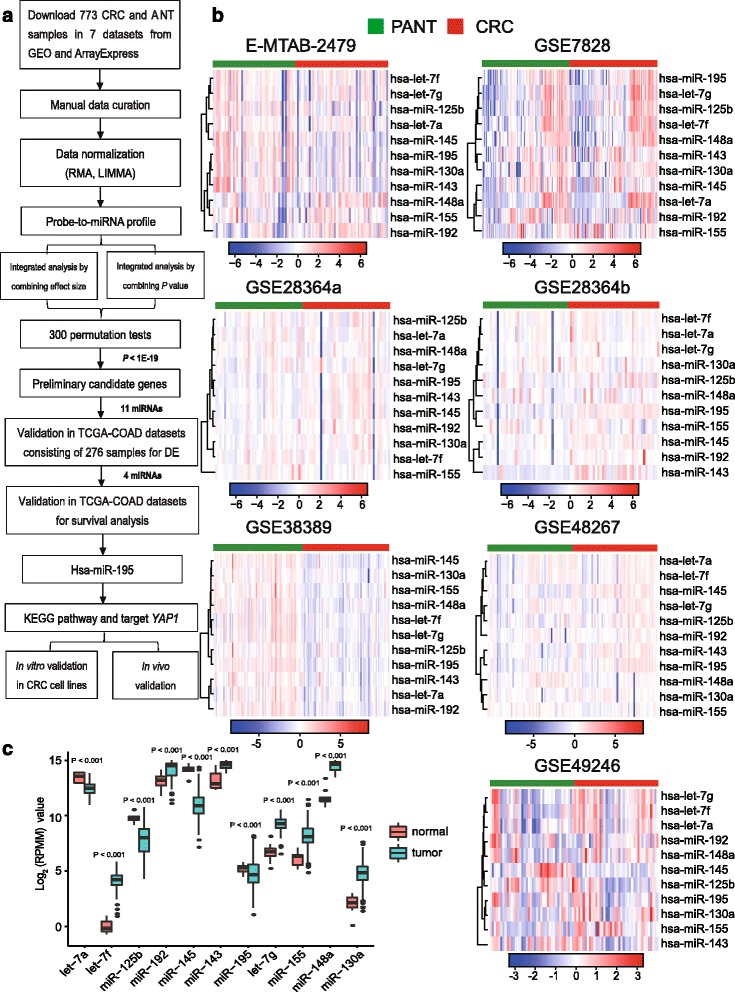
Eleven downregulated genes were identified by integrated analysis of CRC miRNA expression datasets. **a** The flowchart of the integrated analysis and functional validation. *In silico* bioinformatics data analysis pipeline consists of curation of seven publically available datasets, data preprocessing, metaDE and integrated analysis, TCGA dataset validation, validation for diagnostic/prognostic values, KEGG pathway and target gene validation, and in vitro and in vivo functional validation. **b** Clustering of the 11 genes in CRC vs. PANT across each independent dataset. Each *column* represents a sample and each *row* represents the expression level of a given miRNA. The *color scale* represents the raw *Z* score ranging from *blue* (low expression) to *red* (high expression). *Dendrograms* by each heatmap correspond to the hierarchical clustering by expression of the 11 miRNAs. **c** Expression of the downregulated miRNAs is plotted for tumor and normal tissues (TCGA dataset). Expression values of miRNAs are log_2_-transformed

We located and manually curated 23 published and publicly available CRC datasets (see Methods; Additional file 4: Table S2). We identified seven datasets that met the criteria listed in Methods (387 CRC samples and 386 PANT samples; Additional file 5: Table S3). The study selection flow chart for this integrated analysis is shown in Fig. 1a. After merging the datasets, 32 differential miRNAs were identified. Twenty-five miRNAs were retained after filtering by the mean and SD (10%) to filter small expression intensities and small variation genes. We identified 21 miRNAs showing consistent DE patterns using a moderated *t* test by adding a fudging parameter, and Fisher’s method by summarizing -log(*P* value) across studies and running 300 permutations to eliminate significant influence of the large number of samples [28]. One-sided tests revealed 10 upregulated (highly expressed) miRNAs in CRC compared with PANT (Additional file 6: Table S4) and 11 downregulated (lowly expressed) ones in CRC compared with PANT (Additional file 7: Table S5). After combining the effect size, we identified a total of 21 DE miRNAs that showed similar profiles of DE genes to those using Fisher’s method of combining *P* value (*P* < 1E-19; Additional file 8: Table S6). As expected, hierarchical clustering of the seven datasets using the 11 downregulated miRNAs could distinguish the CRC from the PANT samples (Fig. 1b).

### Integrated-signature miRNAs showed clinical prognostic significance in CRC patients

**Fig. 2:**
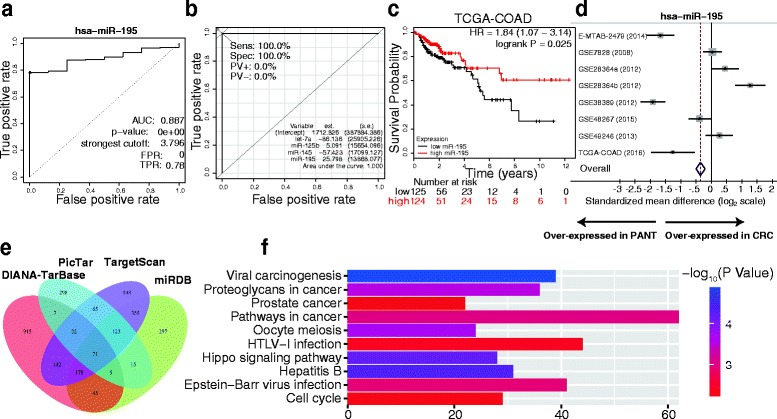
The four validated downregulated miRNAs may serve as significant prognostic markers in CRC classification. **a** An ROC curve built on a univariate classification model based on miR-195 expression across independent TCGA dataset for predicting CRC. **b** Performance of the four miRNAs on CRC tissue classification. CRC risk score was built using a linear regression model by R software. **c** Kaplan–Meier plots for overall survival for a discriminatory median miR-195 expression, from TCGA sequencing data to assess prognostic accuracy. *P* values were calculated using the log-rank test. **d** Forest plots summarizing the downregulation of hsa-miR-195 from eight datasets in the integrated analysis. Each *row* represents a study with standardized mean difference between CRC and normal tissues (*black dot*) and the confidence interval of 95% (*dark line*). The size of the *gray box* is proportional to the relative effect size of each dataset. The *dotted vertical* line at 0 represents the null hypothesis. The *diamonds* represent overall, combined mean difference for miR-195. Thus, negative values indicate downregulation of miR-195 in CRC. **e** Venn diagrams of putative miR-195 targets predicted by TargetScan (v7.1), miRDB, PicTar, and DIANA-TarBase (v7.0). **f** Top ten KEGG pathways that are enriched for the miR-195 targets are mainly cancer-specific pathways

We further validated the 11 DE miRNAs in TCGA-COAD (268 CRC, 8 adjacent normal tissue; Table 1, Additional file 9: Table S7, Fig. 1c). Only hsa-let-7a, hsa-miR-125b, hsa-miR-145, and hsa-miR-195 were significantly downregulated in CRC tumors (Fig. 1c), and each of these miRNAs could provide a high accuracy on CRC tissue classification as estimated by ROC curve analysis (Fig. 2a; Additional file 10: Figure S1a). We optimized the accuracy by using a linear regression model built on a panel of the combined miRNAs (AUC = 1): CRC risk score = 5.091E_miR-125b–57.423E_miR-145–86.136E_let-7a + 25.798E_miR-195 + 1712.826, where E_miR-n = Log _2_ (expression of microRNA) (Fig. 2b).

**Table 1:** Baseline characteristics of patients by miRNA assessment set

	TCGACOAD test cohort	Independent validation cohort
	(*n* = 240)	(*n* = 60)
Gender		
Male	132	38
Female	108	22
Age		
60 years or younger	82	22
Older than 60 years	158	38
TNM stage		
I	39	10
II	93	15
III	75	28
IV	33	7
Pathologic M stage		
M0	207	53
M1	33	7
Nodes count		
12 or more	189	19
Less than 12	51	41
Lymphatic invasion		
Absent	166	25
Present	74	35
Vascular Invasion		
Absent	193	47
Present	47	13
Follow-up time (months)		
Median	13.367	33
Range	(0.067–141.1)	(4.0–57.0)
Preoperative serum CEA level		
<5 μg/mL	188	37
≥5 μg/mL	52	23

Abbreviation:CEA = carcinoembryonicantigen

To further investigate whether the deregulated miRNAs correlate with the survivals of the CRC patients, we performed Kaplan-Meier and Cox’s proportional hazards regression model analysis and found that low miR-195 level was significantly correlated with poor overall survivals of CRC patients (Fig. 2c, Table 2), suggesting the prognostic value of miR-195-5p in clinical CRC diagnosis. The downregulation of miR-195 has been consistently observed in the CRC patients with poor overall survivals, indicating that miR-195 may be functionally important in CRC pathogenesis. In contrast, low levels of let-7, miR-125b, or miR-145 in TCGA CRC tissues were correlated with improved overall survivals (Additional file 10: Figure S1b).

**Table 2:** Univariate and multivariate analyses of clinicopathological characteristics, miR-195-5p, and YAP1 with overall survival in TCGA COAD cohort and independent validation cohort

	Univariate analysis^a^		Multivariate analysis^b^	
	HR (95% Cl)	*P* value	HR (95% CI)	*P* value
TCGA COAD testing set (*n* = 240)				
Gender(male vs. female)	***1.938***(***1.065***–***3.526***)	***0.030***	***2.512***(***1.321***–***4.775***)	***0.005***
Age, years (≥median vs. < median)	1.591(0.843–3.003)	0.152		
Nodes count (12 or more vs. fewer than 12)	0.726(0.402–1.309)	0.287		
Lymphatic invasion (present vs. absent)	***2.171***(***1.238***–***3.809***)	***0.007***	1.453(0.572–3.694)	0.432
Pathologic M stage (present vs. absent)	***3.060***(***1.652***–***5.667***)	***0.001***	***1.969***(***0.988***–***3.924***)	***0.034***
TNM stage (III and IV vs. I and II)	***2.099***(***1.185***–***3.717***)	***0.011***	1.206(0.573–2.536)	0.622
Vascular invasion (present vs*.* absent)	***2.400***(***1.318***–***4.372***)	***0.004***	1.660(0.719–3.830)	0.235
Preoperative CEA level (≥5 μg/mL vs. <5 μg/mL)	0.982(0.542–1.780)	0.953		
Hsa-miR-195 (≥median vs. < median)	***0.488***(***0.262***–***0.910***)	***0.023***	***0.419***(***0.214***–***0.819***)	***0.011***
YAP1 (≥median vs. < median)	***2.022***(***1.120***–***3.652***)	***0.019***	1.379(0.714–2.664)	0.339
Independent validation cohort (*n* = 60)				
Gender(male vs. female)	0.879(0.494–1.564)	0.661		
Age, years (≥median vs. < median)	0.823(0.458–1.478)	0.514		
Nodes count (12 or more vs. fewer than 12)	1.323(0.724–2.418)	0.363		
Lymphatic invasion (present vs*.* absent)	1.542(0.852–2.790)	0.153		
Pathologic M stage (present vs*.* absent)	***16.910***(***5.793***–***49.361***)	***<0.001***	***4.140***(***2.825***–***5.442***)	***0.001***
TNM stage (III and IV vs. I and II)	1.542(0.852–2.789)	0.153		
Vascular invasion (present vs*.* absent)	1.060(0.541–2.075)	0.866		
Preoperative CEA level (≥5 μg/mL vs. <5 μg/mL)	0.893(0.506-1.573)	0.695		
Hsa-miR-195 (≥median vs. < median)	***0.526***(***0.294***–***0.941***)	***0.031***	***0.704***(***0.344***–***0.875***)	***0.044***
YAP1 (≥median vs. < median)	***1.969***(***1.092***–***3.547***)	***0.024***	1.476(0.623–3.497)	0.377

^a^The data were subjected to Cox’s proportional hazards regression model. Bold italics indicate statistically significant values (*P* < 0.05)

^b^Multivariate analysis used stepwise addition and removal of clinical covariates found to be associated with survival in univariate models (*P* < 0.05) and final models include only those covariates that were significantly associated with survival (Wald statistic, *P* < 0.05). Bold italics indicate statistically significant values (*P* < 0.05)

### Functional enrichment and target prediction of miR-195

Significantly, in spite of the presence of biological and technical confounding factors, such as the differences in sample cohorts, treatments, and microarray technologies, in all eight independent CRC expression microarrays, our results indicated the importance of miR-195 expression in human CRC (Fig. 2d). We conducted target prediction for validated miR-195 with high-stringency. Target genes were obtained from both prediction algorithms and experimentally supported databases. The counts of predicted targets, experimentally validated targets, prediction-based consensus targets, and 71 consensus target genes were summarized (Fig. 2e, Additional file 11: Table S8). In addition, we performed KEGG pathway analysis to elucidate the potential biological functions of miR-195 integrated-signature. Interestingly, the top KEGG pathways enriched for the miR-195 targets were mainly associated with cancer-specific pathways (Additional file 12: Table S9), including the Hippo signaling pathway, proteoglycans in cancer, viral carcinogenesis, pathways in cancer, and prostate cancer (Fig. 2f). In previous analysis, the Hippo signaling pathway was significantly enriched for the predicted targets of miR-195-5p (P = 6.47E-05) (Additional file 13: Figure S2). Taken together, these results indicate that deregulated miR-195 expression plays a critical role in human CRC.

### Downregulation of miR-195-5p is concomitant with upregulation of YAP1 in primary human CRC

**Fig. 3:**
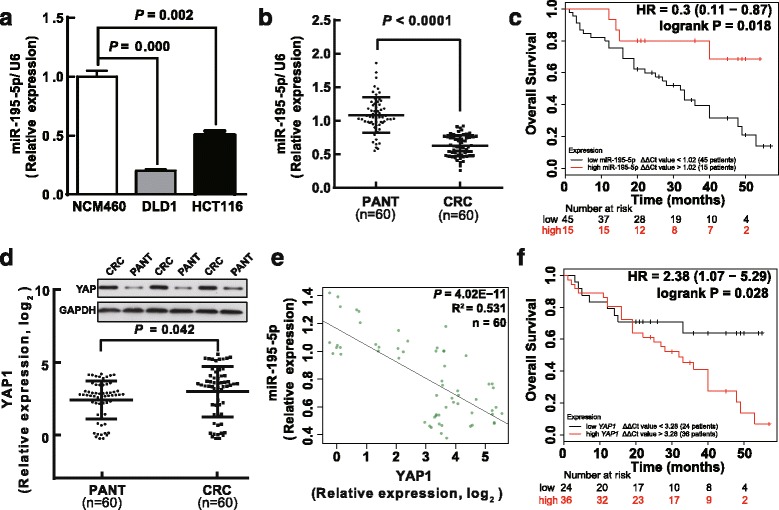
Expressions of miR-195-5p and YAP1 are inversely correlated in primary CRC tumors. **a** Expression of miR-195-5p in two colon cancer cell lines, DLD1 and HCT116, and the normal NCM460 cells. Assays were performed in triplicates. **b** MiR-195-5p is significantly decreased in primary human CRC tissues compared with PANT. Mean ± SD is shown. Statistical analysis was conducted using Student's *t* test. **c** Kaplan–Meier curves of the overall survivals (OS) reveal that downregulated miR-195-5p is associated with poor prognosis in CRC patients. *P* value was obtained by a log-rank test. **d** *YAP1* mRNA and protein levels are shown for the CRC tissues and PANT. **e** Scatter plots showing the inverse association between miR-195-5p and *YAP1* mRNA levels. **f** Kaplan–Meier analysis of OS based on *YAP1* mRNA levels in 60 cases of CRC patients

As miR-195 showed a consistent pattern of downexpression in human CRC, we wondered about the expression pattern of miR-195-5p in the colon cancer cell lines. Similarly, we found a lower expression of miR-195-5p in DLD1 and HCT116 cell lines, compared with the normal intestinal epithelium cell line NCM460 (Fig. 3a). Next, we measured the mature miR-195-5p level in an independent validation sample cohort (*n* = 60) by qRT-PCR (normalized with U6). The results showed that miR-195-5p expression in the tumors was significantly (*P* < 0.05) reduced in the tumors relative to PANT (Fig. 3b). Additionally, Kaplan–Meier and Cox’s proportional hazards regression model survival analysis revealed that patients with low expression levels of miR-195-5p had shorter overall survival (Fig. 3c, Table 2). Thus, given the results of the integrated analysis and the in vitro experiments, we hypothesize that the decreased expression of miR-195-5p may promote CRC progression and development.

Since the Hippo pathway emerged in our previous KEGG analysis for the miR-195 targets, it is conceivable that miR-195 may directly regulate this pathway during CRC tumorigenesis. Meanwhile, the protein-protein interaction network (PPI) suggested that YAP1 is the hub gene of the miR-195 targets (Additional file 14: Figure S3). YAP1 is the pivot in the Hippo signaling pathway leading to potent oncogenicity, and can promote cell growth, migration, invasion, and EMT in many tumors including CRC. Therefore, we also examined YAP1 expression in this sample cohort comprising the 60 human CRC and PANT. Both qRT-PCR and western blot results showed that YAP1 was significantly upregulated in CRC compared with PANT (Fig. 3d). Significantly, the miR-195-5p level was inversely correlated with the YAP1 level in the tumors as calculated by Pearson’s correlation (*R* ^2^ = 0.531, *P* = 4.02E − 11) (Fig. 3e), suggesting that miR-195-5p may downregulate YAP1 in CRC (Fig. 3e). Of note, Kaplan–Meier and Cox’s proportional hazards regression model also revealed that patients with high levels of YAP1 had shorter overall survivals (Fig. 3f, Table 2).

### MiR-195-5p inhibits proliferation and colony formation of colon cancer cells

**Fig. 4:**
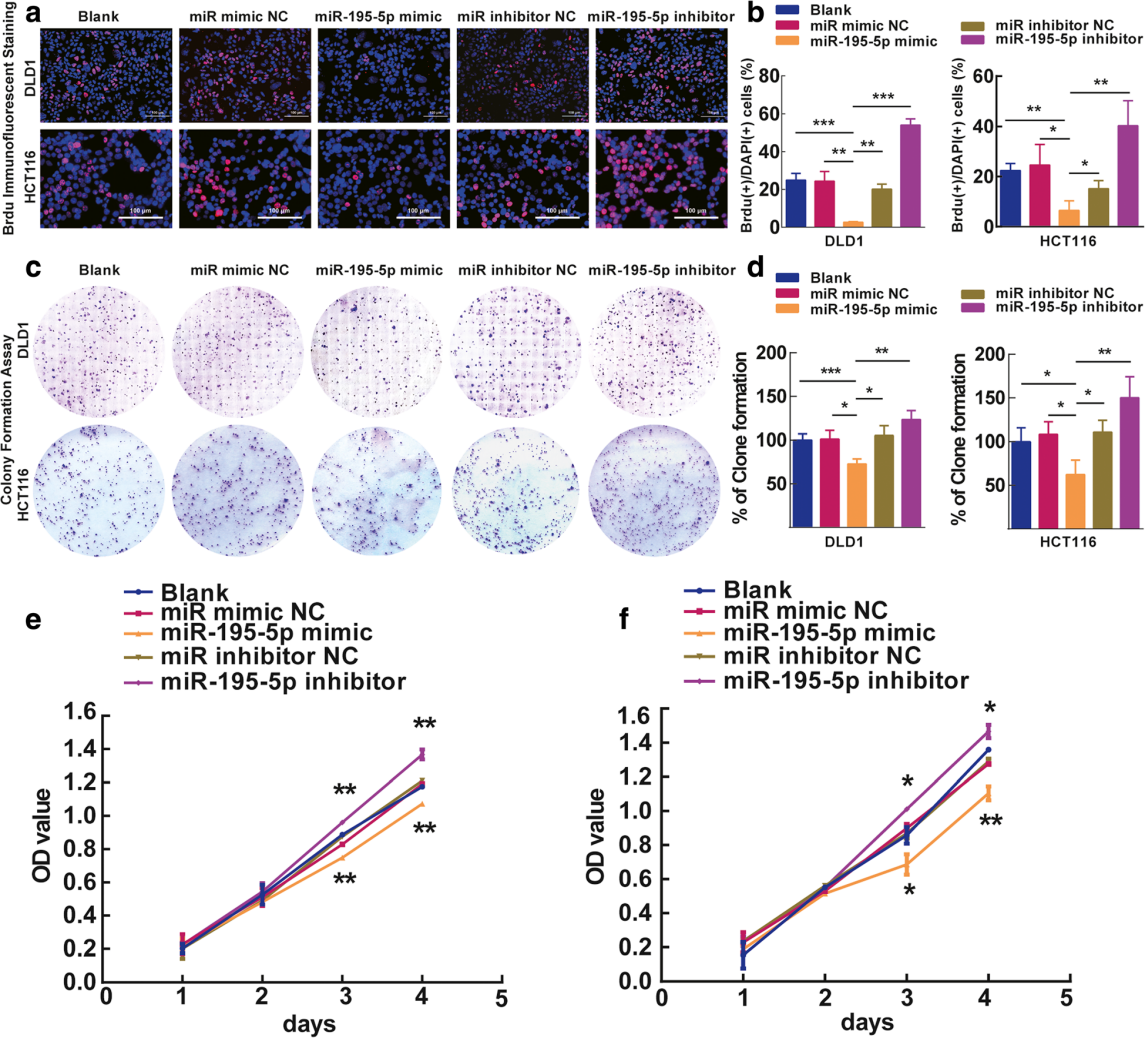
Ectopic expression of miR-195-5p inhibits proliferation and colony formation of DLD1 and HCT116 cells. Representative photomicrographs (**a**) and quantifications (**b**) of BrdU staining in DLD1 and HCT116 cells after transfection with miR-195-5p mimic, miR-195-5p mimic NC, miR-195-5p inhibitor, or miR-195-5p inhibitor NC for 48 h. *Bar* = 100 μm. Representative photomicrographs (**c**) and quantifications (**d**) of the colony formation assay after transfection with miR-195-5p mimic, miR-195-5p mimic NC, miR-195-5p inhibitor, or miR-195-5p inhibitor NC for 8 days. CCK8 cell proliferation assays in DLD1 (**e**) and HCT116 (**f**) cells transfected with indicated miRNAs. Assays were performed in triplicates. Mean ± SD is shown. Statistical analysis was conducted using one-way ANOVA. *P < 0.05. **P < 0.01. ***P < 0.001

We next examined the pathophysiological significance of miR-195-5p downregulation in CRC and its underlying regulatory mechanisms. We transfected DLD1 and HCT116 cells with miR-195-5p mimic or miR mimic NC, and miR-195-5p inhibitor or miR-195-5p inhibitor NC, separately. We first examined the effect of miR-195-5p on proliferation of DLD1 and HCT116 cells. BrdU incorporation assay revealed that miR-195-5p inhibited DNA synthesis in DLD1 and HCT116 cells (Fig. 4a–b). In contrast, miR-195-5p inhibitor treatment could lift this inhibition (Fig. 4a–b). In addition, clonogenic assay showed that miR-195-5p mimic treatment decreased the clonogenic survivals of DLD1 and HCT116 cells compared with blank controls, while miR-195-5p inhibitor-treated DLD1 and HCT116 cells showed a reversed phenotype (Fig. 4c–d), suggesting miR-195-5p negatively regulates cancer cell proliferation. Supporting this notion, a colorimetric based cellular proliferation assay (i.e., CCK8 assay) showed consistent phenotypes when treating with miR-195-5p mimic or its inhibitor (Fig. 4e–f).

### MiR-195-5p inhibits migration and invasion of colon cancer cells

**Fig. 5:**
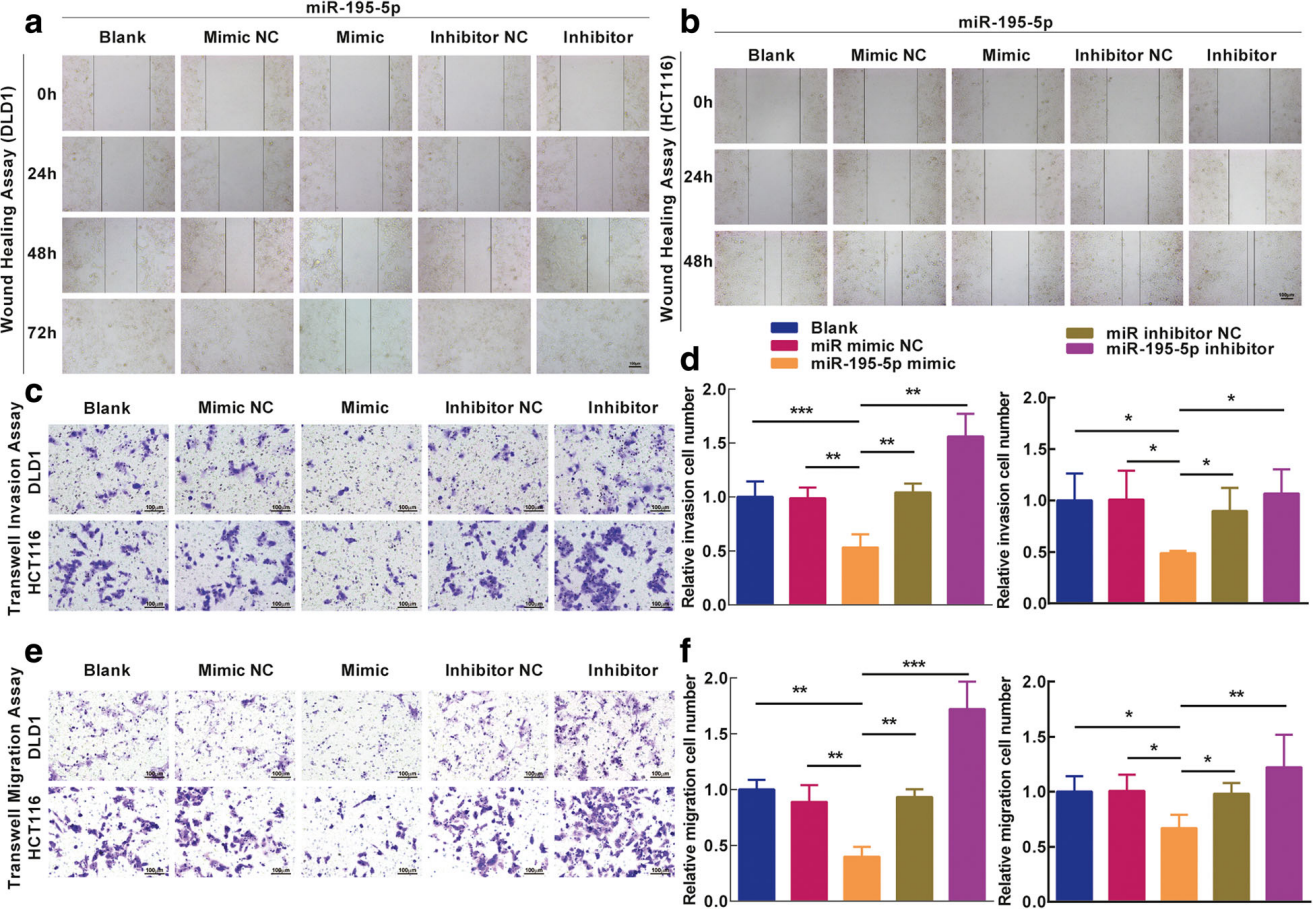
Ectopic expression of miR-195-5p in DLD1 and HCT116 cells reduces cell migration and invasion. **a**–**b** Representative photomicrographs of wound-healing assay in DLD1 and HCT116 cells after transfected miRNAs. *Bar* = 100 μm. **c** Transwell invasion assays of DLD1 and HCT116 cells carrying different miRNAs. *Bar* = 100 μm. **d** Total number of cells in five fields was counted manually. **e** Transwell migration assays of DLD1 and HCT116 cells carrying different miRNAs. *Bar* = 100 μm. **f** Total number of cells in five fields was counted manually. Mean ± SD are shown. Statistical analysis was conducted using one-way ANOVA. *P < 0.05. **P < 0.01. ***P < 0.001

Invasion and migration through the basement membrane are characteristics of metastatic cancer cells. We assessed the role of miR-195-5p on the migration and invasion of DLD1 and HCT116 cells. In the scratch wound healing assay, cell motility of cells was monitored at different time points after generation of the wound. The miR-195-5p-expressing cells migrated toward the wound at a much slower rate than the control or the cells treated by miR-195 inhibitors (Fig. 5a–b). In the transwell invasion and migration assay, we found that invasion and migration of the miR-195-5p-expressing cells was reduced, and this effect could be reversed by the miR-195-5p inhibitor (Fig. 5c–f). These results, taken together, clearly demonstrate that miR-195-5p negatively regulates invasion and migration of colon cancer cells.

### MiR-195-5p targets human YAP1 and inhibits YAP1 in colon cancer cells

**Fig. 6:**
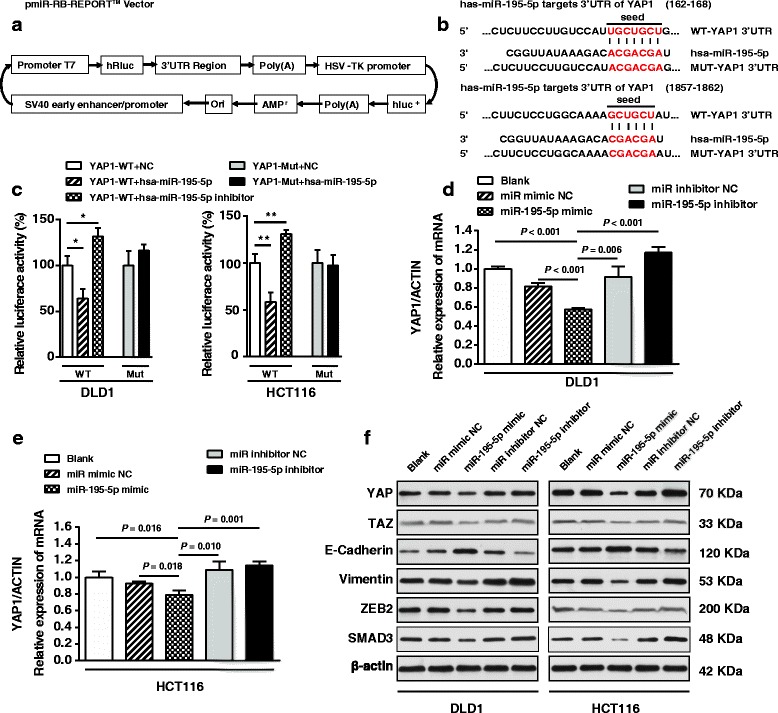
miR-195-5p targets the 3′-UTR of *YAP1* mRNA. **a** A schematic representation of the pmiR-RB-REPORT^TM^ dual luciferase reporter vector. **b** The 3′-UTR of *YAP1* mRNA harbors two miR-195-5p cognate sites. **c** Relative luciferase activity of reporter plasmids carrying wild-type or mutant *YAP1* 3′-UTR in DLD1 and HCT116 cells co-transfected with negative control (NC) or miR-195-5p mimic or miR-195-5p inhibitor. Expression of *YAP1* mRNA in DLD1 (**d**) and HCT116 cells (**e**) transfected with the indicated miRNAs. **f** Western blots of YAP, TAZ, Vimentin, ZEB2, SMAD3, and E-cadherin in DLD1 and HCT116 cells transfected with different miRNAs. Assays were performed in triplicates. Mean ± SD is shown. Statistical analysis was conducted using Student’s *t* test. *P < 0.05. **P < 0.01

To test if YAP1 expression is regulated by miR-195-5p, we cloned the YAP1 3′-UTR into a luciferase reporter plasmid (Fig. 6a) and we quantified expression of the adjacent hRluc coding region. For this purpose, we searched different databases for the potential targets of miR-195-5p that exhibited oncogenic properties. YAP1, which harbors two conserved miR-195-5p cognate sites, namely, 162-168 and 1857–1862 of YAP1 3′-UTR (Fig. 6b), is a predicted target of miR-195-5p. The luciferase reporter plasmid pmiR-RB-REPORT^TM^-YAP1-3′-UTR or mutant reporter plasmid carrying point mutations in the putative miR-195-5p binding sites was co-transfected with miR-195-5p mimics or miR mimic NC and inhibitors, separately. The results showed that miR-195-5p suppressed luciferase activity whereas miR-195-5p inhibitor could promote luciferase activity for the reporter plasmid carrying wild-type YAP1 3′-UTR (Fig. 6c, *P* < 0.05), but no significant effects were observed for the reporter plasmid carrying mutant YAP1 3′-UTR (i.e., pmiR-RB-REPORT^TM^-mut-YAP1-3′-UTR). These results suggest that miR-195-5p binds directly to the predicted binding site(s) in the YAP1 3′-UTR and negatively regulates YAP1 expression.

To confirm the direct regulation of miR-195-5p on YAP1 expression, we transfected DLD1 and HCT116 cells with miR-195-5p mimic, miR mimic NC, miR-195-5p inhibitor, and miR-195-5p inhibitor NC. Both qRT-PCR and western blotting revealed that the YAP1 level was reduced in miR-195-5p-expressing cells, while its level was restored in miR-195-5p inhibitor-treated cells (Fig. 6d–f). The conversion from epithelial cells to mesenchymal cells, characterized by spindle-type cell morphology, was observed in HCT116 cells treated with miR-195-5p inhibitor (Additional file 15: Figure S4). So far, the results suggest that miR-195-5p negatively regulates YAP1 levels in CRC cells. Additionally, expressions of TAZ, Vimentin, ZEB2, and SMAD3 protein were also negatively regulated by miR-195-5p, while E-cadherin was positively regulated by miR-195-5p, suggesting the negative regulations of miR-195-5p on the Hippo pathway and EMT in CRC cells.

### Silence of YAP1 expression inhibits colon cancer cell growth, clone formation, invasion, migration, and rescue assay

We next evaluated the potential tumorigenicity of YAP1 in CRC. Silence of YAP1 expression by siRNA significantly inhibited the expression of YAP (Additional file 16: Figure S5). Moreover, loss of YAP1 expression also contributed to inhibition of colon cancer cell (DLD1 and HCT116 cells) growth (Additional file 16: Figure S5a), clone formation (Additional file 16: Figure S5b–c), invasion, and migration (Additional file 16: Figure S5d–e). These results further verified the powerful tumorigenicity of YAP1 in CRC. These results indicated that the anti-cancer and reverse EMT efficacy of miR-195-5p is partly attributed to its inhibitory role on YAP, which was confirmed by qRT-PCR and western blot of YAP1 (Additional file 16: Figure S5f-g) in DLD1 cells.

### MiR-195-5p suppresses tumor growth in vivo

**Fig. 7:**
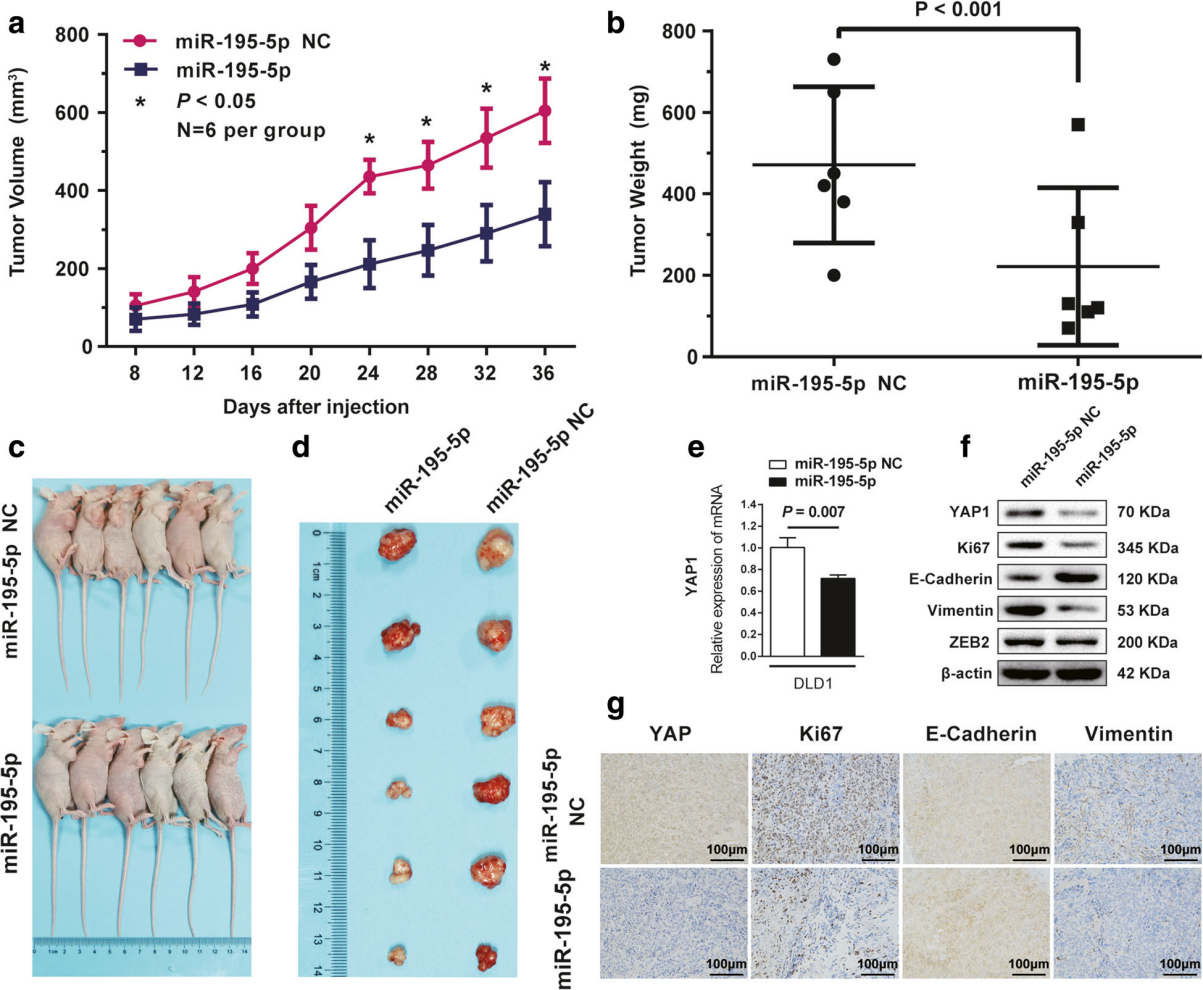
Ectopic expression of miR-195-5p suppresses tumor growth in vivo. The volumes (**a**) and weights (**b**) of the tumors grown in the xenograft mouse model. Data are presented as mean ± SD (*n* = 6); *P < 0.05. Photographs of the mice (**c**) and dissected tumors (**d**) that were treated with or without Agomir-195-5p. **e** Expression of *YAP1* mRNA in DLD1 tumors. **f** Western blots of YAP, Ki67, E-cadherin, vimentin, and ZEB2 in the tumors. β-actin was used as a loading control. **g** Immunohistochemistry of tumor tissues treated with or without miR-195-5p. Assays were performed in triplicates. Mean ± SD is shown. Statistical analysis was conducted using Student’s *t* test.

To confirm the tumor suppressor role of miR-195-5p in vivo, we established a BALB/c nude mouse xenograft model using DLD1 cells. Starting day 8 post-implantation, we injected miR-195-5p agomir or miR agomir NC intratumorally every 4 days for 7 treatments. We evaluated in vivo tumor growth by measuring the tumor volumes and weights. Both the volumes and weights of the tumors treated with miR-195-5p agomir were significantly reduced relative to those treated with miR agomir NC (Fig. 7a–d). Therefore, miR-195-5p significantly inhibits the tumorigenicity of DLD1 cells in vivo. Additionally, consistent with the proposed regulations of miR-195-5p on the Hippo pathway and EMT, expression patterns of YAP, Ki67, Vimentin, ZEB2, and E-cadherin in miR-195-5p-agomir-treated tumors were similar to the in vitro results, substantiating the tumor suppressor function of miR-195-5p in CRC tumorigenesis (Fig. 7e–g).

## Discussion

Integrated analysis, a conscientious approach to unify cross-platform standardization of gene profiling and overlook the unavailability of raw data, has been used to identify differentially expressed genes at the mRNA and miRNA level in CRC [29, 30]. Chen et al. overcame the drawback of lack of conformity among miRNA expression profiling studies in CRC by means of directly combining the ranks of prioritized miRNA lists but not re-analyzing the raw miRNAs profiles [31]. However, they failed to consider the effects of small sample size and heterogeneity of the individual studies because they included 17 studies whose sample size was under 30 pairs in total 24 studies. Conversely, our integrated analysis of the raw data of the seven studies whose sample size is over 30 pairs reliably reveals the differential miRNAs across various platforms.

Our current study supports the validity and effectiveness of this integrated strategy. Firstly, reduced expression of miR-195-5p in primary CRC was revealed independently by both integrated analysis and TCGA-COAD validation analysis, suggesting miR-195-5p can be an effective diagnostic and prognostic marker in the clinical setting. Secondly, ectopic expression of miR-195-5p in colon cancer cell lines considerably decreased cell growth, migration, and invasion. Thirdly, miR-195-5p can directly regulate expression of YAP1 by targeting its 3′-UTR. It can inhibit expression of YAP1, TAZ, Vimentin, ZEB2, and SMAD3, and promote E-cadherin expression in colon cancer cells. Finally, miR-195-5p suppressed tumor growth in vivo.

The goals of the current study were to identify the candidate driver miRNAs in CRC through meta-analysis of multiple datasets of miRNA expression arrays and to validate their biological functions and significance for clinical diagnosis and prognostic prediction. The integrated analysis method described here was designed to exclude the low precision studies, account for biases inherent in single studies, and nominate prevalently dysregulated miRNAs. Taking into account these considerations, we have included seven miRNAs expression data from seven different platforms in CRC using the MetaDE method, which provided options for gene merging, matching across studies, and gene filtering [22]. We screened 10 upregulated and 11 downregulated miRNAs in CRC samples using moderated *t* test and Fisher’s method by summarizing -log(*P* value). In addition, we validated and obtained 4 downregulated miRNAs (i.e., hsa-let-7a, hsa-miR-125b, hsa-miR-145, and hsa-miR-195) from over 250 tumors from the TCGA-COAD database which granted us the opportunity to systematically analyze the potential molecular mechanisms associated with the pathophysiology of CRC. These four miRNAs seem to have a context-specific value as a diagnostic marker of CRC. For hsa-let-7a, hsa-miR-125b, and hsa-miR-145, previous reports have shown their downregulation was correlated with the antitumor effect in CRC [31, 32]. In this study, we found that hsa-let-7a, hsa-miR-125b, and hsa-miR-145 were downregulated in CRC and correlated with improved survivals of CRC patients. Only the low level of miR-195 in tumors was substantially correlated with reduced overall survivals of CRC patients, suggesting miR-195 may negatively regulate CRC progression.

MiR-195 as a tumor suppressor has been reported in various types of cancer. It is a member of paralogous, evolutionarily conserved miRNAs termed the miR-15/107 family that has been suggested to have considerable potential in prognostic prediction [33–38]. Soon et al. found aberrant expression of miR-195 could indicate a poorer prognosis in adrenocortical carcinoma [39]. Li et al. found that extracellular vesicles delivering miR-195 to intrahepatic cholangiocarcinoma could decrease the tumor size and improve survivals in a rat model [40]. Luo et al. found miR-195 might tender a novel tactic for the diagnosis and treatment of breast cancer patients [41]. In our study, we confirmed that miR-195 was downregulated in CRC tissues, which was significantly correlated with poor survivals of CRC patients. We investigated the mechanism of miR-195-5p-mediated regulations on colon cancer cells and found that over-expression of miR-195-5p markedly inhibited cellular proliferation and invasion of CRC cells. Supported by miRNA target analysis and luciferase reporter assays, we propose that miR-195-5p targets the 3′-UTR of *YAP1* mRNA and inhibits YAP expression, leading to proliferative inhibition in CRC cells. Furthermore, the present study also shed light on the potential role of miR-195-5p in CRC metastasis, where ectopic expression of miR-195-5p could reduce cell migration and invasion and promote expression of the EMT marker, E-cadherin. To our knowledge, this is the first meta-analysis that reveals the detailed mechanism where loss of miR-195-5p leads to malignant progression of CRC via unleashed expression of YAP1.

It is well known that Hippo signaling controls cell growth and differentiation [42]. In several tumors, aberrant activation of the Hippo/YAP signaling pathway represents one of the most important mechanisms accounting for oncogenic progression and invasiveness, and has been recognized as a prognostic factor of poor outcome [11, 43–52]. Major signal transducers of the Hippo pathway were proposed as prognostic factors in colorectal cancer [53, 54]. We also identified the Hippo signaling pathway as being enriched in the prediction analysis of the miR-195-5p targets. Experimentally, we verified that the Hippo pathway core effector *YAP1* mRNA 3′-UTR had two conserved miR-195-5p cognate sites. Interestingly, we also found that loss of miR-195-5p accelerated YAP expression and promoted nuclear accumulation of YAP and EMT in vitro [19, 55, 56]. Hence, major players in this pathway may be tightly regulated by miR-195-5p in the colorectum, and miR-195-5p restoration could target YAP/TAZ/EMT pathway to suppress CRC progression. Furthermore, it is likely that other molecules or signaling pathways will be discovered that are also targeted by miR-195-5p in CRC. Future work will focus on revealing additional functions of miR-195-5p in CRC carcinogenesis and progression.

## Conclusions

In summary, we have performed integrated analysis of miRNomes of human normal colorectum and CRC tissues, which provided us rich resources for exploring the roles of miRNAs in CRC. Four downregulated miRNAs (hsa-let-7a, hsa-miR-125b, hsa-miR-145, and hsa-miR-195) can be potentially useful diagnostic markers in the clinic. Among them, only miR-195 is inversely correlated with overall survival in CRC patients. We have demonstrated that miR-195-5p is dramatically downregulated in human CRC tissues compared with normal colorectal tissues. Moreover, upregulation of miR-195-5p suppresses proliferation, migration, invasion, and EMT of colon cancer cells through targeting the *YAP1* mRNA 3′-UTR. Collectively, miR-195-5p suppresses tumor cell growth in vitro and tumorigenicity in vivo. Our study may serve as rational for targeting the miR-195-5p/YAP interaction in a novel therapeutic application to medicate CRC patients.

## Additional files

Additional file 1: **Table S1.** The full-detailed search strategy and searching terms through PubMed, GEO, and Array Express databases. (XLS 26 kb)

Additional file 2: Supplemental R script. The R code for receiver operating characteristic curve analysis. (DOCX 15 kb)

Additional file 3: Supplemental Experimental Procedures. (DOCX 27 kb)

Additional file 4: **Table S2.** The 23 full data sets which were potentially relevant studies assessed for eligibility. Contributor, assay type, platform, source accession, and PMID were listed alongside sample size (normal/tumor). (XLS 34 kb)

Additional file 5: **Table S3.** Datasets used in final quantitative synthesis and integrated analysis. Contributor, assay type, platform, source accession, and PMID were listed alongside sample size (normal/tumor). (XLS 27 kb)

Additional file 6: **Table S4.** Gene-level results in 10 overexpressed differentiated expressed miRNAs in CRC compared with ANT. Statistical measures of integrated analyses are listed. (XLS 24 kb)

Additional file 7: **Table S5.** Gene-level results in 11 underexpressed differentiated expressed miRNAs in CRC compared with ANT. Statistical measures of integrated analyses are listed. (XLS 24 kb)

Additional file 8: **Table S6.** MiRNA-level results in integrated analysis of seven datasets. Statistical measures of integrated analyses are listed. (XLS 32 kb)

Additional file 9 **Table S7.** 11 underexpressed miRNAs validation results in TCGA-COAD dataset. (XLS 96 kb)

Additional file 10: **Figure S1.** The four validated downregulated miRNAs may serve as significant prognostic markers in CRC classification. (A) Receiver operating characteristic (ROC) curve analysis were showed a high performance classification accuracy of CRC tissue and normal tissue in TCGA dataset. (B) Kaplan-Meier survival curves of overall survival in TCGA Cohort according to the ratio of miR-195, let-7a, miR-125b or miR-145 miRNAs level in each tumor sample compared to its control, the median value of this ratio in each cohort was chosen as the cut-off point. (PDF 1081 kb)

Additional file 11: **Table S8.** Seventy-one consensus target genes were summarized by 4 different target prediction algorithms. (XLS 150 kb)

Additional file 12: **Table S9.** Enrichment analysis of predicted miR-195 targets in KEGG cell signaling pathway database. (XLS 25 kb)

Additional file 13: **Figure S2.** KEGG cell signaling pathway was shown for HIPPO pathway. The most significantly enriched by the predicted targets of miR-195 (*P* = 6.47E-05). Red frame shows the predicted miR-195 targets. (TIF 83 kb)

Additional file 14: **Figure S3.** Protein-protein interaction network of the consensus target gene of miR-195-5p. (TIF 2196 kb)

Additional file 15: **Figure S4.** HCT116 cell lines were showed in cell morphology after transfection miR-195-5p inhibitor (10 nM) after 7–10 days. The change in morphology was observed under a light microscope. HCT116 treated with miR-195-5p inhibitor showed mesenchymal features. (TIF 3202 kb)

Additional file 16: **Figure S5.** Silence of YAP1 expression inhibits colon cancer cell growth, invasion, migration and rescue assay. A. CCK8 assays of DLD1 and HCT116 cells after transfected (un-transfected) with siRNA YAP1. B-C. Shown are representative photomicrographs of colony formation assay after transfected with (without) siRNA YAP1 for eight days. D. Shown are representative photomicrographs of transwell invasion assay after transfected with (without) siRNA YAP1. E. Shown are representative photomicrographs of transwell migration assay after transfected with (without) siRNA YAP1. F. Expression of YAP1 mRNA in siRNA YAP1 treated and blank DLD1 cell. G. Western blot of YAP, TAZ, E-cadherin, Vimentin, ZEB2 protein in siRNA YAP1 treated and blank DLD1 cell. Assays were performed in triplicate. Means ± SD are shown. Statistical analysis was conducted using student’s *t*-test. *P < 0.05. **P < 0.01. ***P < 0.001. (TIF 3081 kb)

## Abbreviations

3′-UTR: 3’-untranslated region
ANT: Adjacent normal tissue
COAD: Colon adenocarcinoma
CRC: Colorectal cancer
DE: Differentially expressed
EMT: Epithelial mesenchymal transition
GEO: Gene Expression Omnibus
miR: MicroRNA/miRNA
miR-195: Hsa-microRNA-195
NC: Negative control
PANT: Paired adjacent normal tissue
PPI: Protein-protein interaction network
qRT-PCR: Quantitative reverse transcription polymerase chain reaction
SD: Standard deviation
TAZ: Transcriptional coactivator with PDZ-binding motif
TCGA: The Cancer Genome Atlas
YAP: Yes-associated protein
