# Supplementary material for: Integrated analysis identifies microRNA-195 as a suppressor of Hippo-YAP pathway in colorectal cancer
Source: J Hematol Oncol. 2017 Mar 29;10:79. doi: 10.1186/s13045-017-0445-8 (PMC5372308; doi:10.1186/s13045-017-0445-8)
Supplement: Supplementary file 2 — Supplemental R script. The R code for receiver operating characteristic curve analysis. (DOCX 15 kb) [file 13045_2017_445_MOESM2_ESM.docx]

#----------------------------------------

# ROC analysis script

#----------------------------------------

#

# Supplemental R script for following study:

# Zsuzsanna Mihaly, A meta-analysis of gene expression based biomarkers predicting outcome after tamoxifen treatment in breast cancer

#

# The working directory must contain following files: expr.txt, klin.txt

# Input:

# - gene expression data: expr.txt

# - clinical data: klin.txt

# Output: output_single.txt or output_all.txt files

#

# Example:

rm(list=ls())

getwd()

setwd("”)

#1-------------------------------------------------------------------------------

## try http:// if https:// URLs are not supported

# source("https://bioconductor.org/biocLite.R")

# biocLite("ROC")

# install.packages("ROC")

# install.packages("ROCR")

library(ROC)

library(ROCR)

library(hash)

#2------------------------------------ -------------------------------------------

MyAUC <- function(c, x){

n <- length(c)

n1 <- length(which(c == 1))

n0 <- n - n1

S1 <- cbind(x[which(c == 1)], 1)

S0 <- cbind(x[which(c == 0)], 1)

U <- 0

for( i in 1 : n1){

for(j in 1 : n0){

if(S1[i, 1] > S0[j, 1]) {U <- U + 1}

if(S1[i, 1] == S0[j, 1]) {U <- U + .5}

}

}

auc <- U/(n1 * n0)

vpj <- vector(length=n1)

vqk <- vector(length=n0)

U1 <- 0

for(i in 1 : n1){

for(j in 1 : n0){

if(S1[i, 1] > S0[j, 1]) {U1 <- U1 + 1}

else {if(S1[i, 1] == S0[j, 1]) {U1 <- U1 + 0.50 }}

}

division <- U1 / n0

resta <- (division - auc)^2

vpj[i] <- resta

U1 <- 0

}

U2 <- 0

resta <- 0

for(j in 1 : n0){

for(i in 1 : n1){

if(S1[i, 1] > S0[j, 1]) {U2 <- U2 + 1}

else {if(S1[i, 1] == S0[j, 1]) {U2 <- U2 + 0.50 }}

}

division <- U2 / n1

resta <- (division - auc)^2

vqk[j] <- resta

U2 <- 0

}

vpj <- vpj/(n1 * (n1 - 1))

vqk <- vqk/(n0 * (n0 - 1))

var <- sum(vpj) + sum(vqk)

s <- sqrt(var)

p <- 1 - pnorm((auc - 0.5) / s)

return(c(AUC = auc, lower.95 = qnorm(0.025, mean = auc, sd = s), upper.95 = qnorm(0.975, mean = auc, sd = s), p = p))

}

ROCmain <- function(input1) {

input1 = as.name(input1)

if (input1 == 'ALL') {

a1 <- read.table("klin.txt", header= TRUE, col.names=c("Affyid","status"));

b1 <- read.table("expr.txt", header= TRUE);

# merged <- merge(a1, b1,by="Affyid")

# by = intersect(names(x), names(y))

# merged <- merge(a1, b1,by=intersect(names(a1), names(b1)))

merged <- merge(a1, b1,by="Affyid")

merged1 <- na.omit(merged)

state_vector <- merged1$status

colnames(merged1) = sub("X", "", colnames(merged1), ignore.case =FALSE, fixed=FALSE)

gene_names = colnames(merged1)

index1 = 1:dim(merged1)[2]

index <- 2:dim(merged1)[2]

gene_list <- hash( keys= gene_names, values= index1)

for (j in index) {

marker_vector <- merged1[[j]]

roc <- rocdemo.sca( truth=state_vector, data=marker_vector, rule=dxrule.sca)

auc_value <- AUC(roc)

result <- MyAUC(state_vector, marker_vector);

p_value = result[['p']]

if (auc_value < 0.50){

auc_value = 1 - auc_value

p_value = 1 - p_value

}

res1 = c(as.name(gene_names[j]), auc_value, p_value)

unlist(res1)

res = t(res1);

write.table(res, "output_all.txt", append = TRUE, row.names = FALSE, col.name=FALSE)

}

}

else {

a1 <- read.table("klin.txt", header= TRUE, col.names=c("Affyid","status"));

b1 <- read.table("expr.txt", header= TRUE);

merged <- merge(a1, b1,by="Affyid")

merged1 <- na.omit(merged)

state_vector <- merged1$status

colnames(merged1) = sub("X", "", colnames(merged1), ignore.case =FALSE, fixed=FALSE)

gene_names = colnames(merged1)

index1 = 1:dim(merged1)[2]

index <- 2:dim(merged1)[2]

gene_list <- hash( keys= gene_names, values= index1)

input1 = as.name(input1)

indices = values(gene_list, input1, USE.NAMES = FALSE)

single_gene_index = indices

marker_vector = merged1[[single_gene_index]]

roc <- rocdemo.sca( truth=state_vector, data=marker_vector, rule=dxrule.sca)

auc_value <- AUC(roc)

result <- MyAUC(state_vector, marker_vector);

p_value = result[['p']]

if (auc_value < 0.50){

auc_value = 1 - auc_value

p_value = 1 - p_value

negator = c(rep(1, length(state_vector)))

state_vector = negator - state_vector

roc <- rocdemo.sca( truth=state_vector, data=marker_vector, rule=dxrule.sca)

auc_value <- AUC(roc)

}

auc_value = format(auc_value, digits = 3)

if (p_value < 0.05) {

p_value = format(p_value, digits = 2, scientific = TRUE)

}

else{

p_value = format(p_value, digits = 2, scientific = FALSE)

}

var_filename = as.name(gene_names[single_gene_index])

res1 = c(as.name(gene_names[single_gene_index]), auc_value, p_value)

unlist(res1)

res = t(res1);

write.table(res, file=paste(var_filename, ".txt", sep=""), append = TRUE, row.names = FALSE, col.name=FALSE)

target_pred <- marker_vector

target_class <- state_vector

pred <- prediction(target_pred, target_class)

perf = performance(pred, "acc")

perf1 = performance(pred, "tpr", "fpr")

perf2 = performance(pred, "ppv")

perf3 = performance(pred, "npv")

cutoff.list.acc <- unlist(perf@x.values[[1]])

acc.list <- unlist(perf@y.values[[1]])

optimal_index = which.max(perf@y.values[[1]])

fp_rate = performance(pred, "fpr")

tp_rate = performance(pred, "tpr")

fpr = as.numeric(fp_rate@y.values[[1]])

tpr = as.numeric(tp_rate@y.values[[1]])

dist = rep(0, length(fpr))

dist = as.numeric(dist)

dist_index = length(fpr)

for (k in 1:dist_index){

dist[[k]] <- sqrt(((fpr[[k]])^2) + (((tpr[[k]])-1)^2))

}

best_cutoff_index = which.min(dist)

fpr_opt = fp_rate@y.values[[1]][best_cutoff_index]

tpr_opt = tp_rate@y.values[[1]][best_cutoff_index]

fpr_opt = format(fpr_opt, digits=2)

tpr_opt = format(tpr_opt, digits=2)

optimal.cutoff.acc <- cutoff.list.acc[best_cutoff_index]

p1 = optimal.cutoff.acc

p2 = acc.list[[best_cutoff_index]]

perf2@y.values[[1]][is.na(perf2@y.values[[1]])] = 0

perf3@y.values[[1]][is.na(perf3@y.values[[1]])] = 0

#i1 = integrate(as.numeric(perf2@y.values[[1]]), min(perf2@x.values[[1]]), max(perf2@x.values[[1]]))

#i2 = integrate(as.numeric(perf2@y.values[[1]]), min(perf2@x.values[[1]]), max(perf2@x.values[[1]]))

x = c(0.0, 0.1, 0.2, 0.3, 0.4, 0.5, 0.6, 0.7, 0.8, 0.9, 1.0)

y = c(0.0, 0.1, 0.2, 0.3, 0.4, 0.5, 0.6, 0.7, 0.8, 0.9, 1.0)

x1 = cutoff.list.acc

y1 = rep(c(0.5), each = length(cutoff.list.acc))

pdf(file=paste(var_filename, ".pdf", sep=""))

plot(roc, main = as.name(gene_names[single_gene_index]), xlab ="False positive rate", ylab = "True positive rate")

points(fpr_opt, tpr_opt)

text(0.8, 0.25, "AUC:", font = 2)

text(0.9, 0.25, auc_value)

text(0.78, 0.2, "p-value:", font = 2)

text(0.92, 0.2, p_value)

text(0.715, 0.15, "strongest cutoff:", font = 2)

text(0.9, 0.15, optimal.cutoff.acc)

text(0.8, 0.1, "FPR:", font = 3)

text(0.9, 0.1, fpr_opt)

text(0.8, 0.05, "TPR:", font = 3)

text(0.9, 0.05, tpr_opt)

#auc_value, "p-value:", p_value, "strongest cutoff:", "cutoff value:", optimal.cutoff.acc, "FPR:", fpr_opt, "TPR:", tpr_opt),pch=NA, bty="n")

lines(x, y, type = "l", lty=2, lwd=1)

#lines(x1, y1, type = "l", lty=2, lwd=1)

#plot(perf2@x.values[[1]], perf2@y.values[[1]], type="l", col="red", xlab ="Cutoffs", ylab = "Pos/Neg predictive value", ylim=c(0,1))

#par(new=TRUE)

#plot(perf2@x.values[[1]], perf3@y.values[[1]], main = as.name(gene_names[single_gene_index]), type="l", col="green", xlab="", ylab="")

#legend("bottomright", c("positive predictive values","negative predictive values"), lty=c(1,1), col=c("red", "green"))

dev.off()

}

}

#3------------------------------------ --------------------------------------------

ROCmain('ALL')

ROCmain('Expr')

#
