# Supplementary material for: Integrated analysis identifies microRNA-195 as a suppressor of Hippo-YAP pathway in colorectal cancer
Source: J Hematol Oncol. 2017 Mar 29;10:79. doi: 10.1186/s13045-017-0445-8 (PMC5372308; doi:10.1186/s13045-017-0445-8)
Supplement: Supplementary file 3 — Supplemental Experimental Procedures. (DOCX 27 kb) [file 13045_2017_445_MOESM3_ESM.docx]

**Integrated analysis identifies microRNA-195 as a suppressor of Hippo-YAP pathway in colorectal cancer**

**RUNNING TITLE: MicroRNA-195 targeting Hippo/YAP pathway in colorectal cancer**

Min Sun, Haibin Song, Shuyi Wang, Chunxiao Zhang, Liang Zhen, Fangfang Chen, Dongdong Shi, Yuanyuan Chen, Chaogang Yang, Zhenxian Xiang, Qing Liu, Chen Wei, Bin Xiong

**Supplemental Experimental Procedures (SEP)**

**Dual luciferase reporter assays**

The 3′-untranslated region (UTR) of human YAP1 were amplified from human genomic DNA and individually inserted into the pmiR-RB-REPORT^TM^ (Ribobio, Guangzhou, China) using the Xhol and Notl sites. Similarly, the fragment of YAP1 3′-UTR mutant was inserted into the pmiR-RB-REPORT^TM^ control vector at the same sites. For reporter assays, HCT116 and DLD1 cells were co-transfected with wild-type (mutant) reporter plasmid and miR-Ribo^TM^ mimics (miR-Ribo^TM^ negative control) using Lipofectamine 2000 (Invitrogen). Firefly and Renilla luciferase activities were measured in cell lysates using the Dual-Luciferase Reporter Assay system. Luciferase activity was measured forty-eight hours post-transfection using dual-glo luciferase reporter system according to the manufacturer's instructions (Promega, Madison, WI, USA). Firefly luciferase units were normalized against Renilla luciferase units to control for transfection efficiency.

**BrdU immunofluorescence assay**

DLD1 and HCT116 cells were seeded on sterile cover glasses placed in the 6-well plates. After transfection with miR-195-5p mimic, miR mimic NC, miR-195-5p inhibitor, miR inhibitor NC for forty eight hours, the BrdU (5-bromo-2-deoxyuridine; Sigma) stock solution at 10 mg/mL in saline was diluted 1000× in the culture medium and incubated for 60 min. After washing with PBS, cells were then fixed for 20 min in 4% paraformaldehyde (PFA) and permeabilized with 0.3% Triton X-100 for 10 min. After blocking with 10% goat serum in PBS for 1h, cells were incubated with a primary rabbit antibody against BrdU (1:200, Abcam) over night at 4°C, and then incubated with the secondary antibody coupled to a fluorescent marker, Cy3, at room temperature for 2 h. After DAPI staining and PBS washing, the cover slips were mounted on to glass slides with anti-fade solution and visualized using a fluorescence microscope (Olympus 600 auto-biochemical analyzer, Tokyo, Japan) with Image-Pro Plus software for image analysis, and 5 microscopic fields were taken for calculating BrdU.

**Colony formation assay**

Cells were transfected with miR-195-5p mimic or miR mimic NC, miR-195-5p inhibitor or miR inhibitor NC, as described above. Twenty four hours later, transfected cells were trypsinized, counted, replated at a density of 800 cells/6 cm dish. Eight days later, colonies resulting from the surviving cells were fixed with 3.7% methanol, stained with 0.1% crystal violet and counted. Colonies containing at least 50 cells were scored. Each assay was performed in triplicates.

**CCK8 assay**

Cell growth was measured using the cell proliferation reagent WST-8 (Roche Biochemicals, Mannheim, Germany). After plating cells in 96-well microtiter plates (Corning Costar, Corning, NY) at 1.0 × 10^3^/well, 10 μL of CCK8 was added to each well at the time of harvest, according to the manufacturer's instructions. One hour after adding CCK8, cellular viability was determined by measuring the absorbance of the converted dye at 450 nm.

**Wound healing assay** ***in vitro***

DLD1 and HCT116 cells were seeded in 6-well plates and incubated for twenty four hours, a linear wound was created by dragging a 200μL pipette tip through the monolayer prior to transfection. Cellular debris was removed by gentle washes with culture medium, following which transfection was performed immediately, and the cells were allowed to migrate for a further seventy two hours. The healing process was dynamically photographed after the wound was introduced using a microscope (Olympus 600 auto-biochemical analyzer, Tokyo, Japan). Migration distance was measured from images (5 fields) taken at each indicated time point. The gap size was analyzed using Image-Pro Plus 6.0 software. The residual gap between the migrating cells from the opposing wound edge was expressed as a percentage of the initial gap size.

**Transwell migration/invasion assay**

DLD1 and HCT116 cells were grown in DMEM medium containing 10% fetal bovine serum to ~50% confluence and transfected with 50 nM miR-195-5p mimic or a NC, 100 nM miR-195-5p inhibitor or a NC. After twenty four hours, the cells were harvested by trypsinization and washed once with Hanks’ balanced salt solution (Invitrogen). To measure cell migration, 8-mm pore size culture inserts (Transwell; Costar, High Wycombe, UK) were placed into the wells of 24-well culture plates, separating the upper and the lower chambers. In the lower chamber, 500 μL of DMEM medium containing 10% FBS was added. Then, serum-free medium containing 5 ×10^4^ cells were added to the upper chamber for migration assays, whereas 1 ×10^5^ cells were used for matrigel invasion assays. After forty eight hours of incubation at 37°C with 5% CO_2_, the number of cells that had migrated through the pores was quantified by counting 8 independent visual fields under the microscope (Olympus) using *a* ×200 magnifications, and cell morphology was observed by staining with 0.1% crystal violet. Each experiment was performed at least three times.

**Western blot analysis**

Western blot was completed as described formerly [[1](#_ENREF_1)]. Seventy-two hours after transfection, total protein was extracted from the DLD1 and HCT116 cells using RIPA cell lysis reagent containing proteinase and phosphatase inhibitors (Sangon Biotech, Shanghai, China) at 4°C for 30 min. Cell lysates were centrifuged at 12,000 × g for 10 min at 4°C, and the protein concentrations of the supernatant were determined using the BCA protein assay reagent kit (Aspen). The supernatants containing total protein were then mixed with a corresponding volume of 5 × SDS loading buffer and RIPA cell lysis reagent and then heated at 100°C for 10 min. The supernatant lysates were run on 10% SDS-polyacrylamide gels (50 μg/lane), and proteins were transferred to poly (vinylidene fluoride) (PVDF) membranes (Hertfordshire, UK) by semidry electroblotting (1.5 mA/cm^2^). PVDF membranes were then incubated in blocking buffer [Tris-buffered saline (TBS) supplemented with 0.05% (vol/vol) Tween 20; TBST] containing 5% (wt/vol) skimmed milk powder for 120 min at room temperature followed by three 10 min washes in TBST. The PVDF membranes were then incubated with anti-YAP (1:1000 dilutions, CST), anti-TAZ (1:1,000 dilutions, Affinity), anti-ZEB2(1:1,000 dilutions, Millipore), anti-E-cadherin (1:1,000 dilutions, Affinity), anti-Vimentin (1:1,000 dilutions, Affinity), anti-β-Actin (1:5,000 dilutions, SCB) and GAPDH (1:5,000 dilutions, CST) as internal normalizers in TBST containing 5% (wt/vol) skimmed milk powder (antibody buffer) overnight at 4°C on a three-dimensional rocking table. Then the membranes were washed three times for 10 min in TBST and then incubated with goat anti-rabbit IgG conjugated to horseradish peroxidase (1:4,000 dilutions) in antibody buffer for 180 min. Finally, membranes were washed three times for 8 min in TBST and exposed to Clarity Western ECL Substrate (Bio-Rad Laboratories, Hercules, CA) for 1-2 min as described in the manufacturer's protocol, as described previously [[1](#_ENREF_1)]. The molecular weights of the bands were calculated by a comparison with prestained molecular weight markers (molecular weight range: 6,500 –250,000) that were run in parallel with the samples. Semiquantitative analysis of specific immunolabeled bands were performed using AlphaEaseFC 4.0 (Alpha Innotech).

Table SEP1: Primer sequences for quantitative reverse transcription (RT)-PCR (miRNA and gene) in this study

| **miRNA** | **Sequence (5′ → 3′)** |
| --- | --- |
| **miR-195-5p** | Reverse-transcribed primer:  5'-GTCGTATCCAGTGCAGGGTCCGAGGTATTCGCACTGGATACGACGCCAA-3′ |
|  | Forward: 5'-GTCTAGCAGCACAGAAATA-3'  Reverse: 5'-GTGCAGGGTCCGAGGT-3' |
| **U6** | Reverse transcribed Primer:5′-AACGCTTCACGAATTTGCGT-3′ |
|  | Forward:5′-CTCGCTTCGGCAGCACA-3′  Reverse:5′-AACGCTTCACGAATTTGCGT-3′ |
| **Gene** | **Sequence (5′ → 3′)** |
| **Human YAP1**  **NM_001130145.2** | Forward:5′-TGAGAACAATGACGACCAATAGC-3′  Reverse:5′-TTCAAGGTAGTCTGGGAAACGG-3′ |
| **Human ACTIN**  **NM_001101** | Forward:5′-CACCCAGCACAATGAAGATCAAGAT-3′  Reverse:5′-CCAGTTTTTAAATCCTGAGTCAAGC-3′ |

**Table SEP2: The information of antibodies used in this study**

| Antibodies | Company | Product Number | Molecular Weight (kDa) |
| --- | --- | --- | --- |
| β-actin | Santa Cruz Biotechnology  (Dallas, TX, USA) | sc-1616r | 42 |
| YAP | Cell Signaling Technology  (Beverly, MA, USA ) | 14074 | 65-75 |
| TAZ | Affinity  (Cincinnati, OH, USA) | DF4653 | 33 |
| E-Cadherin | Affinity  (Cincinnati, OH, USA) | AF0131 | 120 |
| Vimentin | Affinity  (Cincinnati, OH, USA) | AF0292 | 53 |
| ZEB2 | Merck Millipore  (Darmstadt, Germany) | abt332 | 200 |
| GAPDH | Cell Signaling Technology  (Beverly, MA, USA ) | 2118 | 37 |
| Ki67 | Santa Cruz Biotechnology  (Dallas, TX, USA) | sc-7846 | 395/345 |

**Table SEP3: The information of dual luciferase reporter assays and transfection reagents used in this study**

| **Reagent** | Product Number | Company |
| --- | --- | --- |
| micrON^TM^ has-miR-195-5p mimic | miR10000870 | Ribobio, Guangzhou, China |
| micrON^TM^ mimic Negative Control | miR01101 | Ribobio, Guangzhou, China |
| micrOFF^TM^ has-miR-195-5p inhibitor | miR20000461 | Ribobio, Guangzhou, China |
| micrOFF^TM^ inhibitor Negative Control | miR02101 | Ribobio, Guangzhou, China |
| micrON^TM^ has-miR-195-5p agomir | miR40000461 | Ribobio, Guangzhou, China |
| micrON^TM^ agomir Negative Control | miR04201 | Ribobio, Guangzhou, China |
| Si-h-YAP1_001 | siB11218131429 | Ribobio, Guangzhou, China |
| Si-h-YAP1_002 | siG000010413B | Ribobio, Guangzhou, China |
| Si-h-YAP1_003 | siG000010413C | Ribobio, Guangzhou, China |
| NControl_05815 | siN05815122147 | Ribobio, Guangzhou, China |
| NControl_05815(cy3) | siN05815122149 | Ribobio, Guangzhou, China |
| Bulge-Loop^TM^ hsa-miR-195-5p RT Primer | ssD809230216 | Ribobio, Guangzhou, China |
| Bulge-Loop^TM^ hsa-miR-195-5p Forward Primer | ssD809230908 | Ribobio, Guangzhou, China |
| Bulge-Loop^TM^ miR-Reverse Primer | ssD089261711 | Ribobio, Guangzhou, China |
| Bulge-Loop^TM^ U6-RT Primer | ssD0904071008 | Ribobio, Guangzhou, China |
| Bulge-Loop^TM^ U6-Forward Primer | ssD0904071006 | Ribobio, Guangzhou, China |
| Bulge-Loop^TM^ U6-Reverse Primer | ssD0904071007 | Ribobio, Guangzhou, China |
| pmiR-RB-Report_h-YAP1(110-2364) | GUR101729 | Ribobio, Guangzhou, China |
| pmiR-RB-Report_h-YAP1(110-2364)  2Mut(TGCTGCT-ACGACGA,GCTGCT-CGACGA) | GUR101730 | Ribobio, Guangzhou, China |
| pmiR-RB-Report_h-YAP1(110-2364),Glycerol Stock | GUR101729G | Ribobio, Guangzhou, China |
| pmiR-RB-Report_h-YAP1(110-2364)  2Mut(TGCTGCT-ACGACGA,GCTGCT-CGACGA),Glycerol Stock | GUR101730G | Ribobio, Guangzhou, China |

***References***

1. Wang Y, Fang W, Huang Y, Hu F, Ying Q, Yang W, Xiong B: Reduction of selenium-binding protein 1 sensitizes cancer cells to selenite via elevating extracellular glutathione: a novel mechanism of cancer-specific cytotoxicity of selenite. Free Radic Biol Med. 2015, 79:186-196.
