# Supplementary figures and images for: Integrated analysis identifies microRNA-195 as a suppressor of Hippo-YAP pathway in colorectal cancer
Source: J Hematol Oncol. 2017 Mar 29;10:79. doi: 10.1186/s13045-017-0445-8 (PMC5372308; doi:10.1186/s13045-017-0445-8)

**A**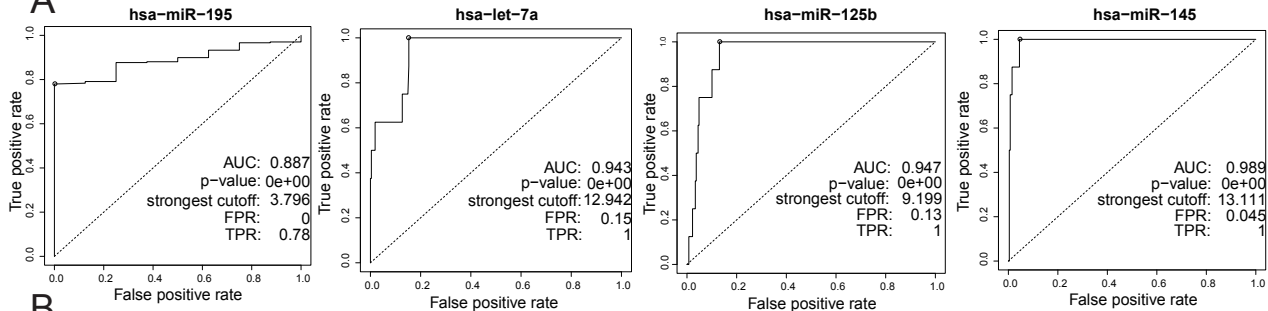**B**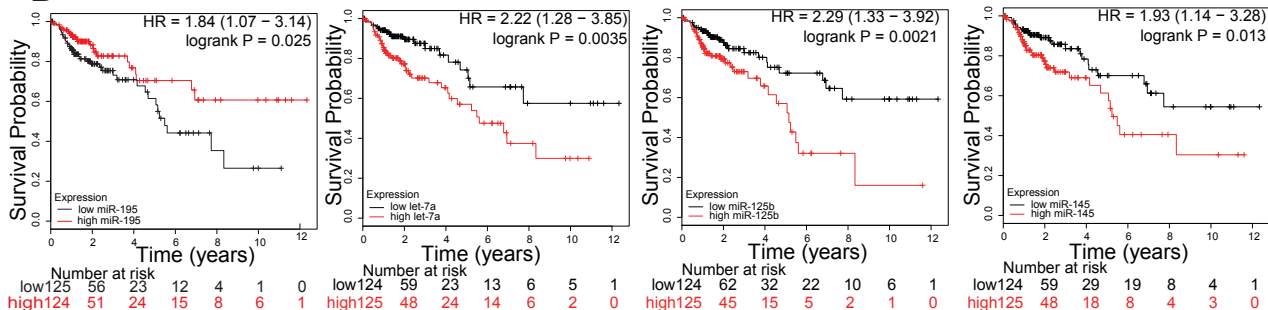

Supplement: Supplementary file 10 — Figure S1. The four validated downregulated miRNAs may serve as significant prognostic markers in CRC classification. (A) Receiver operating characteristic (ROC) curve analysis were showed a high performance classification accuracy of CRC tissue and normal tissue in TCGA dataset. (B) Kaplan-Meier survival curves of overall survival in TCGA Cohort according to the ratio of miR-195, let-7a, miR-125b or miR-145 miRNAs level in each tumor sample compared to its control, the median value of this ratio in each cohort was chosen as the cut-off point. (PDF 1081 kb) [file 13045_2017_445_MOESM10_ESM.pdf]

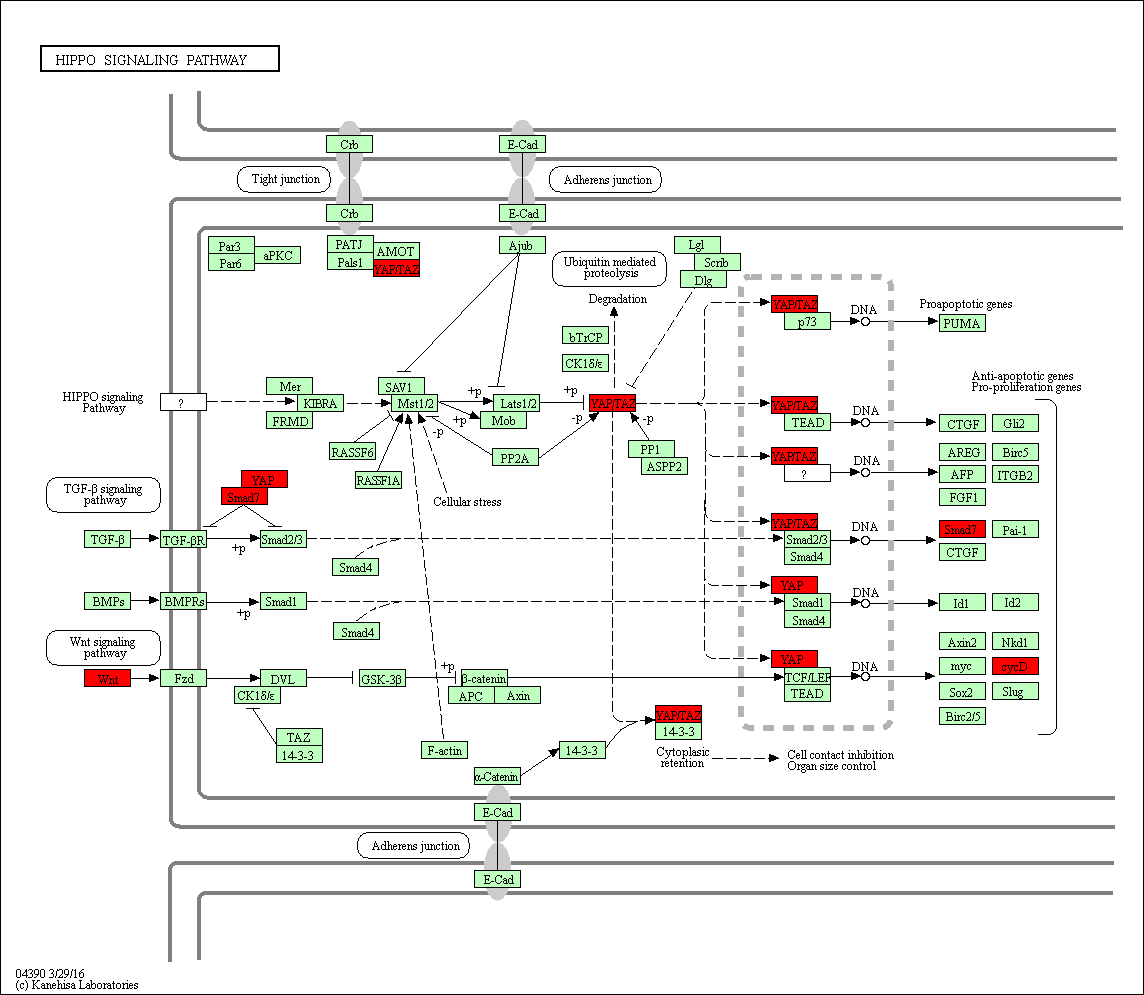

Supplement: Supplementary file 13 — Figure S2. KEGG cell signaling pathway was shown for HIPPO pathway. The most significantly enriched by the predicted targets of miR-195 (P = 6.47E-05). Red frame shows the predicted miR-195 targets. (TIF 83 kb) [file 13045_2017_445_MOESM13_ESM.tif]

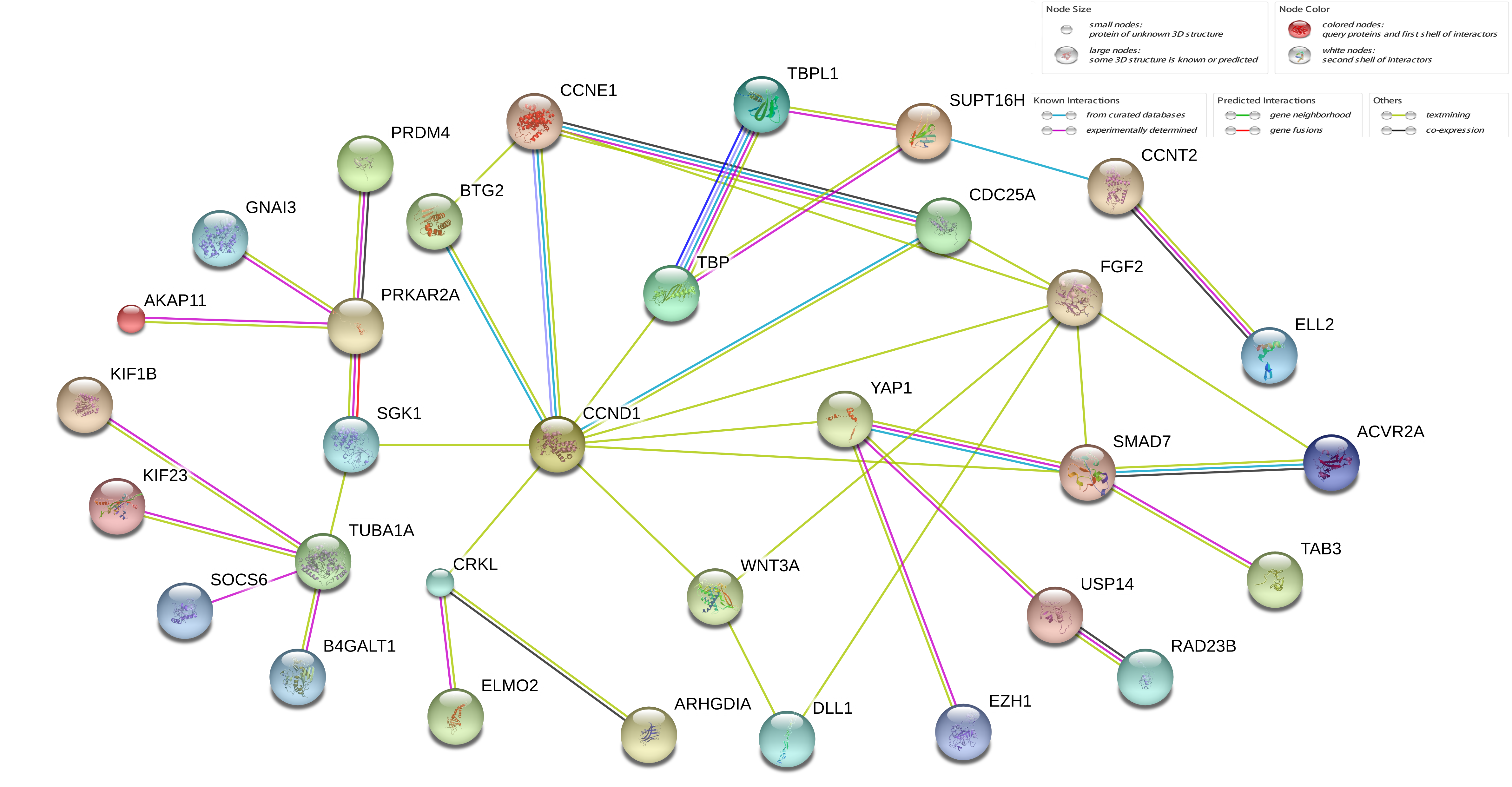

Supplement: Supplementary file 14 — Figure S3. Protein-protein interaction network of the consensus target gene of miR-195-5p. (TIF 2196 kb) [file 13045_2017_445_MOESM14_ESM.tif]

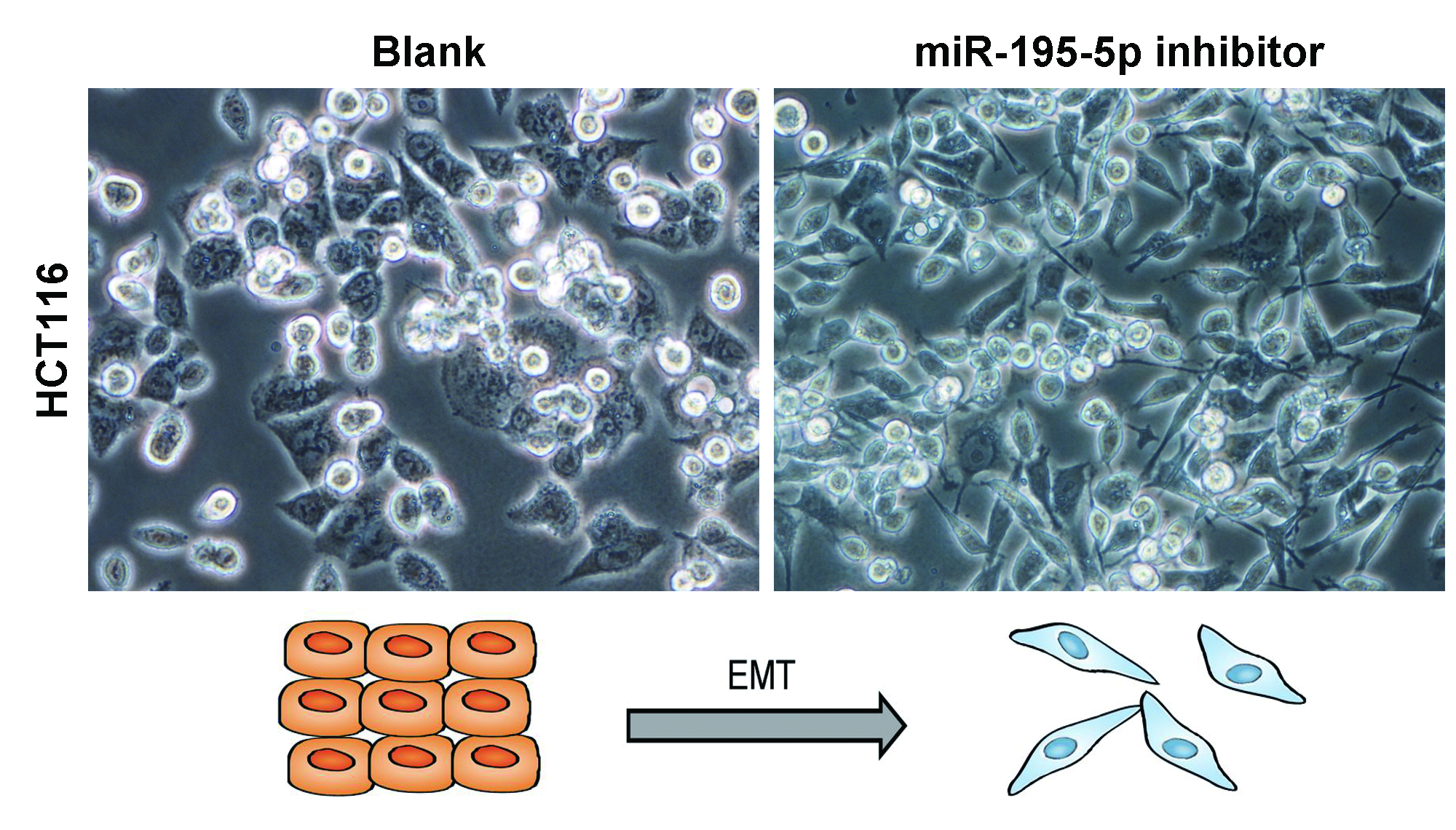

Supplement: Supplementary file 15 — Figure S4. HCT116 cell lines were showed in cell morphology after transfection miR-195-5p inhibitor (10 nM) after 7–10 days. The change in morphology was observed under a light microscope. HCT116 treated with miR-195-5p inhibitor showed mesenchymal features. (TIF 3202 kb) [file 13045_2017_445_MOESM15_ESM.tif]

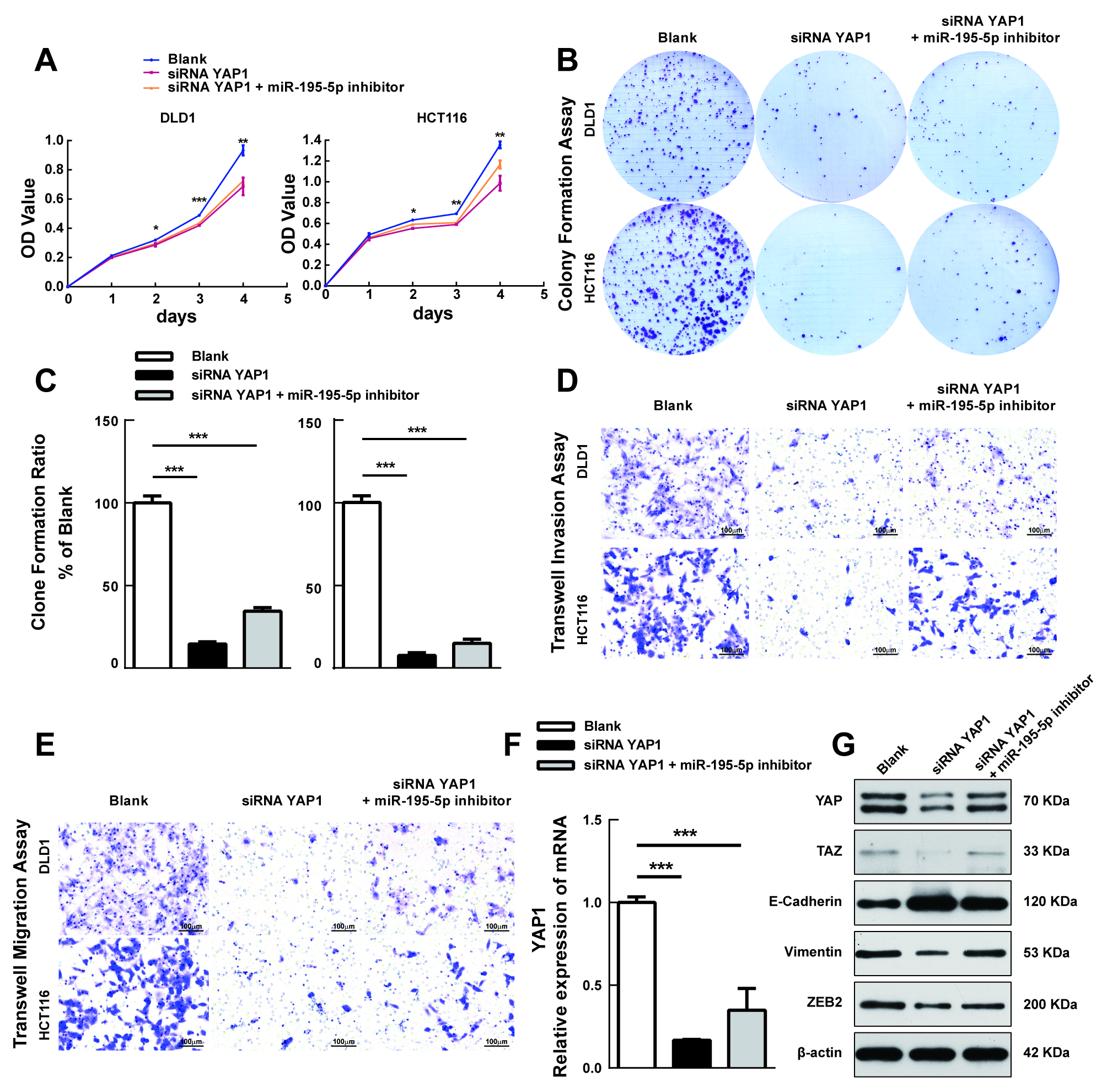

Supplement: Supplementary file 16 — Figure S5. Silence of YAP1 expression inhibits colon cancer cell growth, invasion, migration and rescue assay. A. CCK8 assays of DLD1 and HCT116 cells after transfected (un-transfected) with siRNA YAP1. B-C. Shown are representative photomicrographs of colony formation assay after transfected with (without) siRNA YAP1 for eight days. D. Shown are representative photomicrographs of transwell invasion assay after transfected with (without) siRNA YAP1. E. Shown are representative photomicrographs of transwell migration assay after transfected with (without) siRNA YAP1. F. Expression of YAP1 mRNA in siRNA YAP1 treated and blank DLD1 cell. G. Western blot of YAP, TAZ, E-cadherin, Vimentin, ZEB2 protein in siRNA YAP1 treated and blank DLD1 cell. Assays were performed in triplicate. Means ± SD are shown. Statistical analysis was conducted using student’s t-test. *P < 0.05. **P < 0.01. ***P < 0.001. (TIF 3081 kb) [file 13045_2017_445_MOESM16_ESM.tif]
